# Neocortical Axon Arbors Trade-off Material and Conduction Delay Conservation

**DOI:** 10.1371/journal.pcbi.1000711

**Published:** 2010-03-12

**Authors:** Julian M. L. Budd, Krisztina Kovács, Alex S. Ferecskó, Péter Buzás, Ulf T. Eysel, Zoltán F. Kisvárday

**Affiliations:** 1 School of Informatics, University of Sussex, Brighton, United Kingdom; 2 Department of Neurophysiology, Ruhr-Universität Bochum, Bochum, Germany; 3 Neuronal Networks Group, College of Medical & Dental Sciences, School of Clinical & Experimental Medicine, University of Birmingham, Birmingham, United Kingdom; 4 Institute of Physiology, Medical School, University of Pécs, Pécs, Hungary; 5 Department of Anatomy, Histology & Embryology, University of Debrecen, Debrecen, Hungary; RIKEN Brain Science Institute, Japan

## Abstract

The brain contains a complex network of axons rapidly communicating information between billions of synaptically connected neurons. The morphology of individual axons, therefore, defines the course of information flow within the brain. More than a century ago, Ramón y Cajal proposed that conservation laws to save material (wire) length and limit conduction delay regulate the design of individual axon arbors in cerebral cortex. Yet the spatial and temporal communication costs of single neocortical axons remain undefined. Here, using reconstructions of *in vivo* labelled excitatory spiny cell and inhibitory basket cell intracortical axons combined with a variety of graph optimization algorithms, we empirically investigated Cajal's conservation laws in cerebral cortex for whole three-dimensional (3D) axon arbors, to our knowledge the first study of its kind. We found intracortical axons were significantly longer than optimal. The temporal cost of cortical axons was also suboptimal though far superior to wire-minimized arbors. We discovered that cortical axon branching appears to promote a low temporal dispersion of axonal latencies and a tight relationship between cortical distance and axonal latency. In addition, inhibitory basket cell axonal latencies may occur within a much narrower temporal window than excitatory spiny cell axons, which may help boost signal detection. Thus, to optimize neuronal network communication we find that a modest excess of axonal wire is traded-off to enhance arbor temporal economy and precision. Our results offer insight into the principles of brain organization and communication in and development of grey matter, where temporal precision is a crucial prerequisite for coincidence detection, synchronization and rapid network oscillations.

## Author Summary

Within the grey matter of cerebral cortex is a complex network formed by a dense tangle of individual branching axons mostly of cortical origin. Yet remarkably when presented with a barrage of complex, noisy sensory stimuli this convoluted network architecture computes accurately and rapidly. How does such a highly interconnected though jumbled forest of axonal trees process vital information so quickly? Pioneering neuroscientist Ramón y Cajal thought the size and shape of individual neurons was governed by simple rules to save cellular material and to reduce signal conduction delay. In this study, we investigated how these rules applied to whole axonal trees in neocortex by comparing their 3D structure to equivalent artificial arbors optimized for these rules. We discovered that neocortical axonal trees achieve a balance between these two rules so that a little more cellular material than necessary was used to substantially reduce conduction delays. Importantly, we suggest the nature of arbor branching balances time and material so that neocortical axons may communicate with a high degree of temporal precision, enabling accurate and rapid computation within local cortical networks. This approach could be applied to other neural structures to better understand the functional principles of brain design.

## Introduction

Brains, like electronic networks, face a hard design problem: how to pack very many, yet highly interconnected, discrete computing devices within the least possible space while simultaneously preserving efficient communication [1]. Neocortex, for example, is densely packed and composed mostly of axonal and dendritic ‘wire’ [2],[3] originating largely from massive intracortical interconnectivity [4]–[6]. Each intracortical axon arbor, which can extend over many millimetres, transmits electrical activity from one neuron to thousands of others in its vicinity [6]–[8]. Therefore, each arbor represents a network design problem with at least two distinct communication costs (e.g. [9]): the amount of wire used to connect with all its postsynaptic targets (spatial or construction cost, in the sense of network design), and the time taken for an action potential radiating from the presynaptic cell to reach these targets (temporal or routing cost).

Ramón y Cajal [10] proposed that distinct laws conserving material or ‘wire’ (space), conduction delay (time), and brain volume govern neuronal design, and that from these laws physiological inferences could be made. However, Ramón y Cajal [10],[11] did not attempt to quantify the relative importance of these conservation laws nor how these distinct laws might interact to reproduce neuronal morphology. In recent years, attention has concentrated on material conservation as proposed in the ‘wiring minimization principle’ [12]–[16], which alone is claimed to explain many key features of brain organization including the intracortical wiring underlying functional maps in neocortex [14],[16]. Yet whether intracortical axonal trees in grey matter conform to the wiring minimization principle remains empirically untested and its consequences on temporal cost have not been explicitly considered. Here, we empirically investigated, to our knowledge for the first time, the spatial and temporal cost optimality of whole three-dimensional intracortical axon arbors.

## Results

We investigated the spatial (wire length) and temporal economy of nineteen intracortical axon arbors obtained from in vivo labelling of cat visual cortex. Using detailed single axon reconstructions, we first mapped the three-dimensional (3D) arrangement of axonal boutons produced by each arbor to determine the position of presumptive synaptic connections (fixed vertices) and the parent cell body (root vertex). We then used the axonal tree skeleton to construct a graphical representation of each arbor measuring the direct distance between connected morphological landmarks (cell body, axon bifurcations, and boutons) to obtain wire lengths (edges) (see Figure S1). Next, we used graph optimization algorithms to find both separately and together the spatial and temporal cost minimized arbors representing the same geometry of axon connectivity. Comparing axonal trees against such artificial arbors optimized for spatial and/or temporal cost permitted us to infer how these two distinct requirements shape axonal tree morphology. The results suggest that by using more wire than necessary intracortical axonal arbors ensure that conduction times are typically less than twice the minimum delay and preserve a low degree of temporal dispersion.

### Axon Database

**Figure 1:**
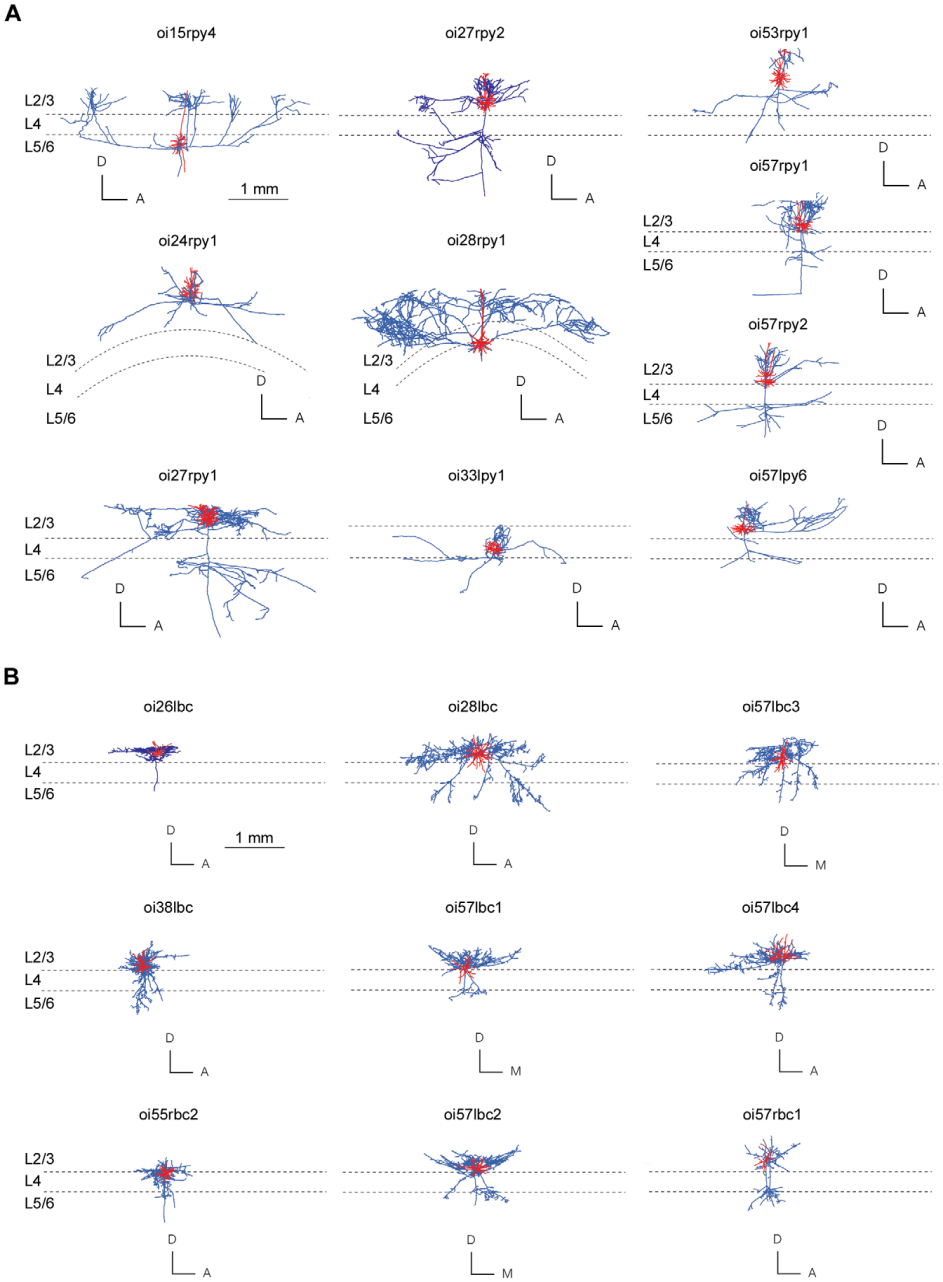
Morphology of spiny and basket cells analysed in study. (A) Spiny cell axons (blue lines) and dendritic (red lines) arbors of each neuron shown in coronal plane against approximate laminar boundaries (n = 10). (B) Basket cell axons (blue lines) and dendritic (red lines) arbors of each neuron shown in coronal plane aligned with approximate laminar boundaries (n = 9). Cell identifiers matched with results given in Table S1. For clarity, axonal boutons are not shown. (Anatomical axes: A, anterior; D, dorsal; M, medial).

Mammalian neocortex is composed of two main morphological classes of neuron: spiny (∼80% of all neurons) and smooth or sparsely-spinous (∼20%) [17]–[20]. We examined intracortical axon arbors of ten putative excitatory (morphologically-identified spiny stellate and pyramidal cells) and nine putative inhibitory cells (morphologically-identified large basket cells), which we three-dimensionally reconstructed after labelling in vivo in adult cat primary visual cortex and are described in more detail elsewhere [21]–[23]. Pyramidal and spiny stellate axons target the dendrites of spiny and smooth neurons [3], [6], [24]–[28], while basket cell axons target the soma and proximal dendrities of both smooth and spiny neurons [8], [20]–[22]. Together these neurons are representative of the majority (85–90%) of neuronal types present in cat visual cortex [6],[8],[18],[29] and their axons are thought to form the long-range networks underlying functional maps [6]–[8], [21]–[23],[30]. The morphology of spiny cell and basket cells analysed here are shown in relation to cortical lamina in Figure 1.

### Optimization Criteria

To test for spatial cost minimization, we used a *minimum spanning tree* (MST) algorithm [31] to find the least amount of wire required to connect together axon origin with all boutons present in a given axon arbor [12]. Total wire length may be further shortened if additional (Steiner) vertices (akin to axon bifurcations **–** nodal point where the axon divides to produce at least two child branches) are inserted to produce an *Euclidean Steiner minimal tree* (ESMT) [32]. However, Steiner tree problems are considered computationally intractable Non-deterministic Polynomial time (NP)-hard [32], so we used the only available (heuristic) algorithm for finding large vertex set ESMT [33], which has proved successful with other 3D datasets. Wire length economy (ε) was calculated from the ratio of minimum to actual total axon wire length.

To test for temporal cost minimization, we approximated temporal cost from the total distance travelled (path length) by a notional axon potential from the axon origin to each bouton. Here the minimum-cost graph is a *star tree*, a single-source shortest path tree with a parallel branch from axon origin to each bouton vertex [9]. To estimate axonal latency, we divided path length by a uniform conduction velocity (see Methods). Axon conduction velocity varies with axon thickness, branching, ion channel density and variety, and myelination [34], so latency estimates here are approximations only. Yet realistic numerical simulations of intracortical axon arbors suggest path length is the main determinant of latency [35]. Current estimates of mean intracortical axonal conduction velocity in adult cat visual cortex vary (range = 0.1–0.6 m s^−1^ [36]–[38]) but are typically slower than, for example, the main type of thalamic afferent axon innervating visual cortex (e.g. X-type geniculate axons, range = 8–20 m s^−1^ [39]). Path length economy (γ) was computed from the ratio of minimum to actual average path length, though similar results were obtained for total path length.

**Figure 2:**
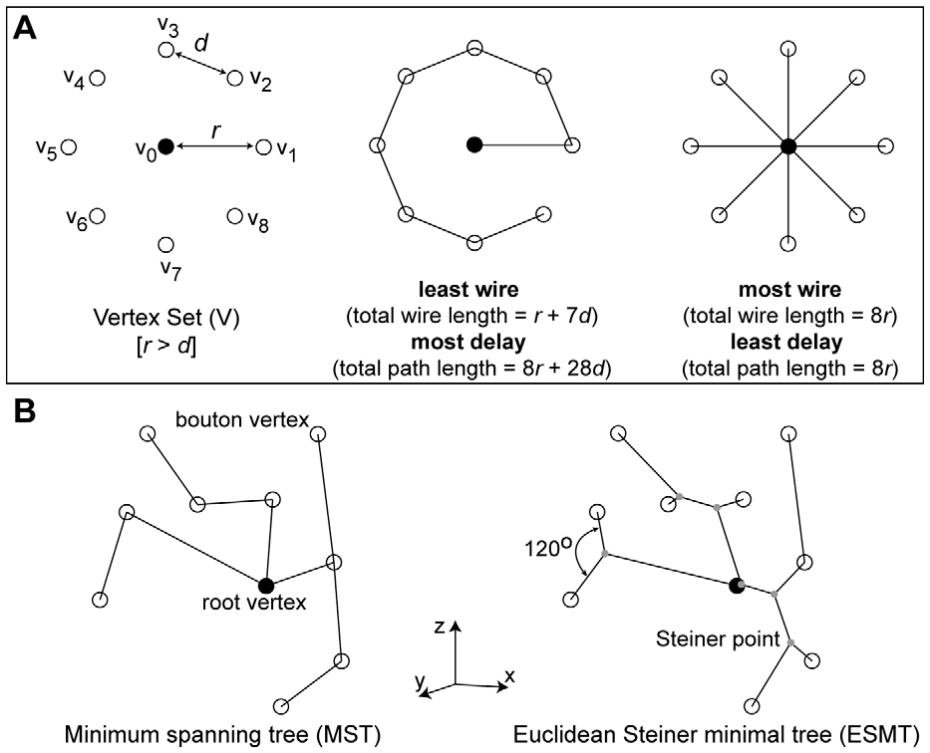
Artificial arbors were used to examine axonal tree optimization. (A) Illustration of artificial arbors minimizing spatial (*middle*) and temporal (*right*) communication cost for a planar ring arrangement of bouton vertices (open circle) surrounding a cell body (filled circle) (*left*). For example, path *p* from cell body (*v_0_*) to bouton (*v_5_*) is *p*(*v_0_*, *v_5_*) = <*v_0_*,*v_1_*,*v_2_*,*v_3_*,*v_4_*,*v_5_*> and <*v_0_*,*v_5_*>, respectively, with corresponding path lengths *d_T_*(*v_0_*,*v_5_*) = *r*+4*d* and *r*. Note any given edge may be an element in more than one path but for wire length an edge is counted once only. (B) A simple 3D problem to illustrate the difference between minimum spanning tree (MST, *left*), where spatial cost is minimized using root and bouton vertices only, and Euclidean Steiner Minimal Tree (ESMT, *right*), where additional vertices called Steiner points (grey dots) may be inserted to further shorten total arbor length provided the interior angle between adjacent vertices and the Steiner point is 120°.

A simple example illustrating the distinction between wire and path length cost minimization [9],[31] is shown in Figure 2. Here, connecting nearby boutons in serial fashion to minimize wire length tends to increase time delay (Figure 2A, middle), whereas dedicating parallel branches to each bouton to minimize time delay dramatically increases wire length (Figure 2A, right). The difference between a 3D MST and an ESMT is shown in Figure 2B. We now consider whether this relationship extends to biological axon arbors and whether biological axons are wire-length optimized.

### Spatial Cost of Axon Arbors

**Figure 3:**
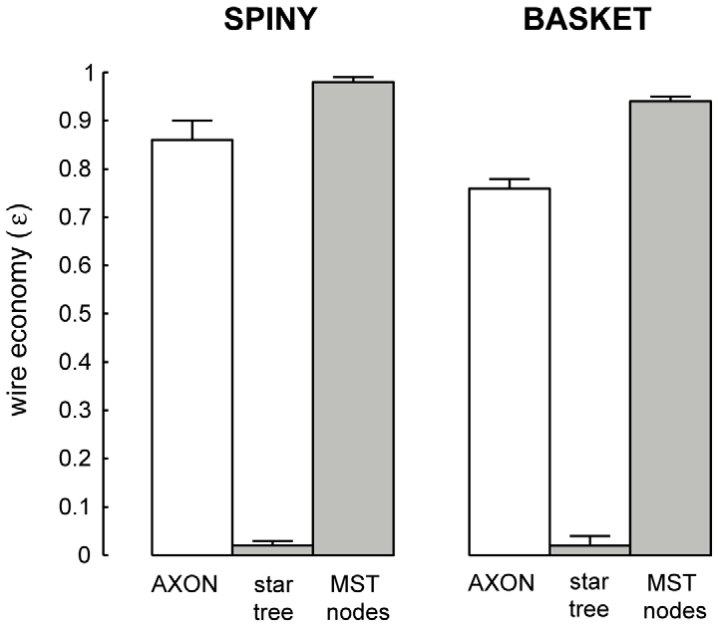
Wire length economy of individual spiny and basket cell intracortical axon arbors was suboptimal. Wire length economy (ε) of spiny and basket cell intracortical axon arbors (*ε_AXON_* = *L_MST_*/*L_AXON_*, where *L_AXON_* is total axon arbor length based on direct distances between boutons) compared with path length optimized star graphs (*ε_STAR_* = *L_MST_*/*L_STAR_*) and MST with additional vertices from axon bifurcations or nodes (*ε_MSTnodes_* = *L_MST_*/*L_MSTnodes_*).

**Figure 4 pcbi-1000711-g004:**
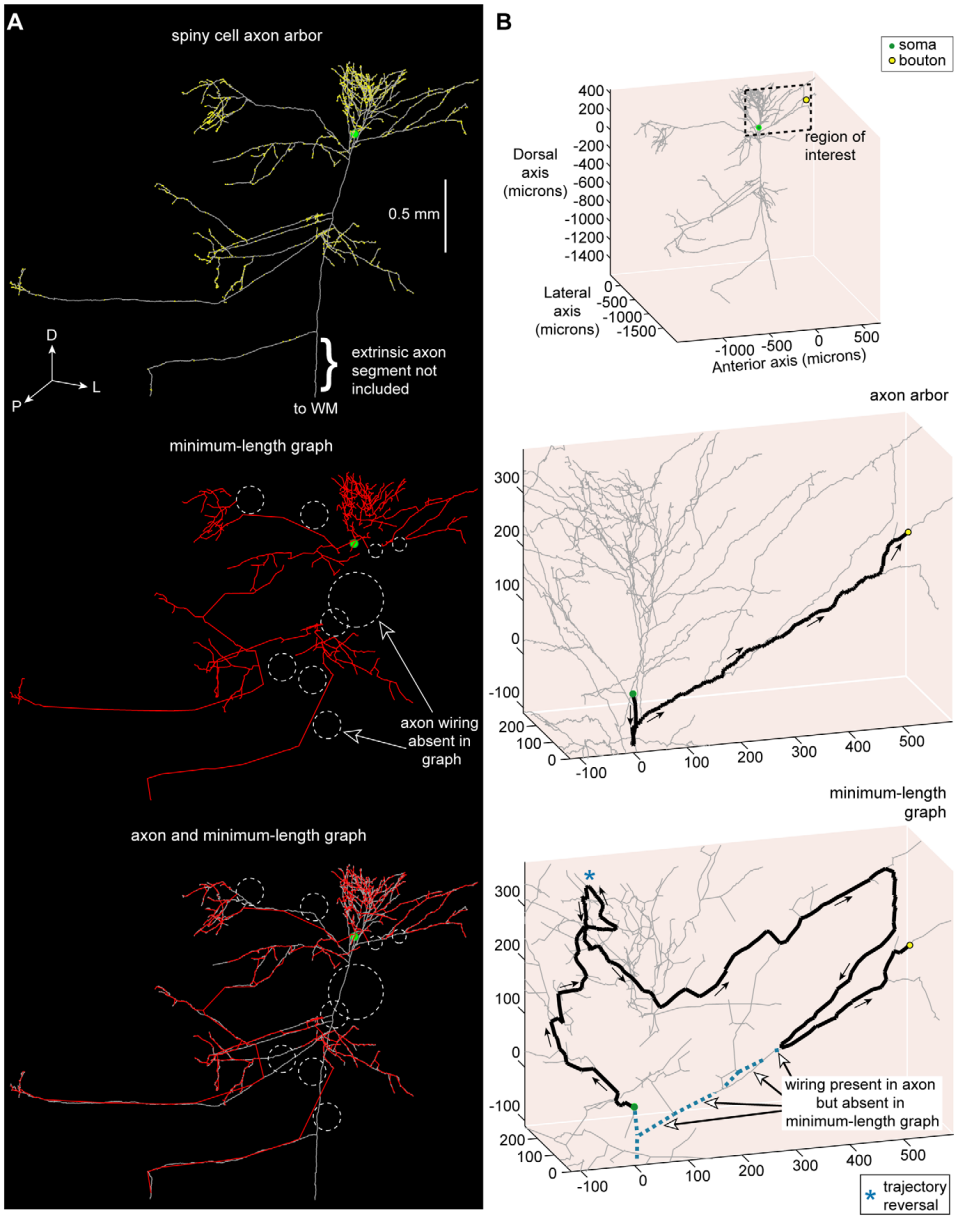
Spiny cell axon arbor wiring compared with minimum-length tree. (A) Example putative excitatory pyramidal cell axon arbor (coronal view) showing the location of numerous boutons (*upper*), its Euclidean Steiner Minimal Tree (ESMT) graph (*middle*), and overlay of axon arbor and graph (*lower*) with dotted circles (white) showing locations where axon wiring was absent in minimum-length graph taken to connect same bouton set. (Key: axon wiring  =  grey lines, graph wiring  =  red lines, axonal bouton  =  yellow dots, cell body  =  green dot; anatomical axes: D, dorsal; L, lateral; P, posterior.) (B) Example of the shortest path from axon origin (root vertex) of this neuron to a selected bouton (*upper*, see region of interest) for the biological arbor (*middle*) was, after branching from the main descending axon, fairly direct (0.85 mm path length) but for the length-minimized tree (*lower*) the route was more circuitous (2.63 mm path length), including a trajectory reversal (marked by blue asterisk), because the artificial arbor lacked wire present in the axon arbor (dotted blue lines). Arrows show direction of flow from axon origin to bouton. (Key: shortest path  =  thick black lines, unvisited arbor wiring  =  grey lines, axon wiring absent in graph  =  dotted blue lines.).

**Figure 5 pcbi-1000711-g005:**
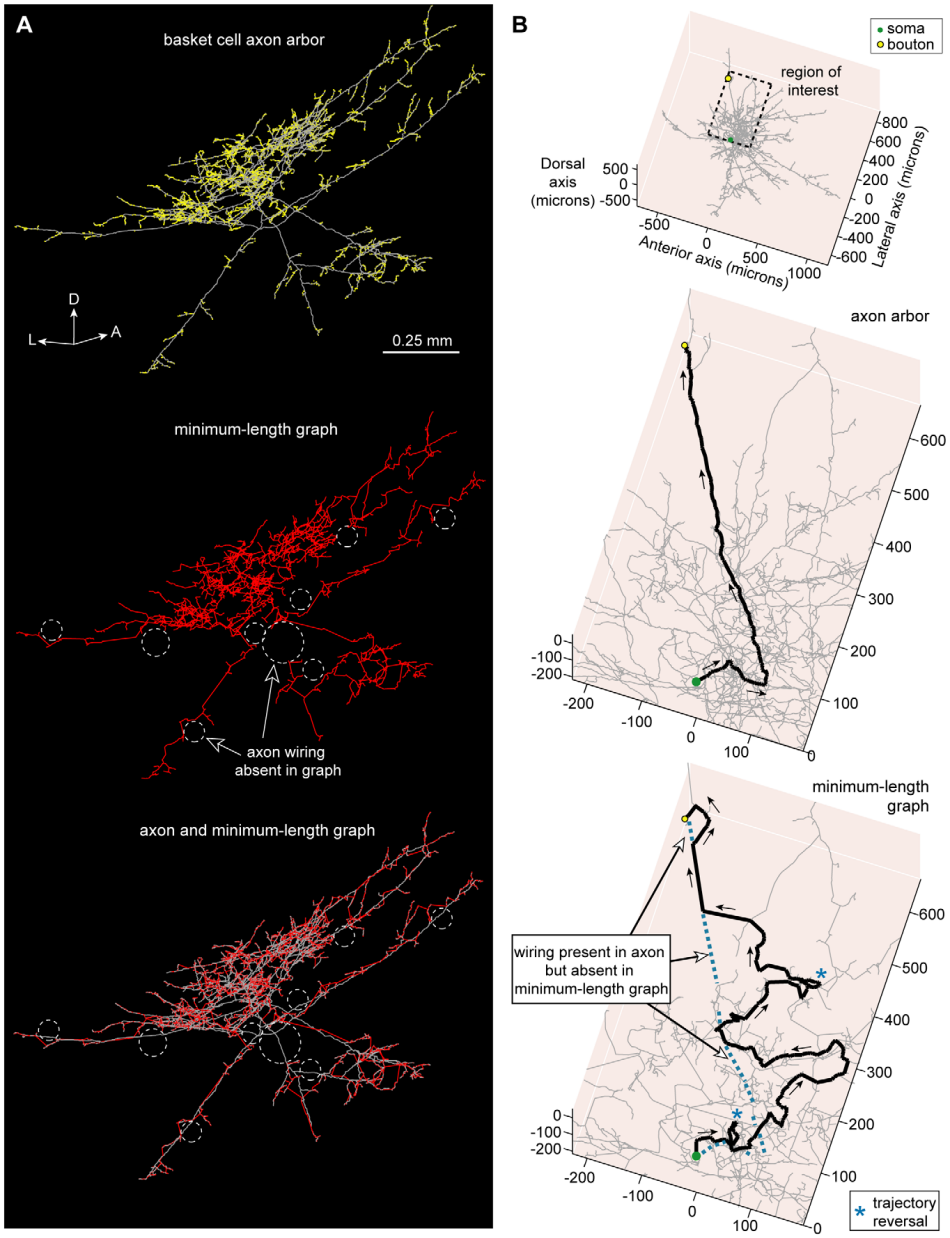
Basket cell axon arbor wiring compared with minimum-length tree. (A) Example putative inhibitory large basket cell axon arbor (coronal view) showing bouton locations (*upper*), its Minimum Spanning Tree (MST) graph (*middle*), and overlay of axon arbor and graph (*lower*) demonstrating different wiring patterns used to connect same bouton set as shown by dotted circles (white). (Key: axon  =  grey lines, graph  =  red lines, boutons  =  yellow dots; anatomical axes: A, anterior; D, dorsal; L, lateral; P, posterior). (B) Example of the shortest path from axon origin (root vertex) of this neuron to a selected bouton (*upper*, see region of interest) for the biological arbor (*middle*) was initially directed away from the bouton but virtually direct thereafter (0.87 mm path length) yet for the length-minimized tree (*lower*) the course was tortuous (2.28 mm path length), including two trajectory reversals (see blue asterisk), because the artificial arbor lacked wire present in axon arbor (dotted blue lines). Arrows show direction of flow from axon origin to bouton. (Key: shortest path  =  thick black lines, unvisited arbor wiring  =  grey lines, axon wiring absent in graph  =  dotted blue lines.).

**Figure 6 pcbi-1000711-g006:**
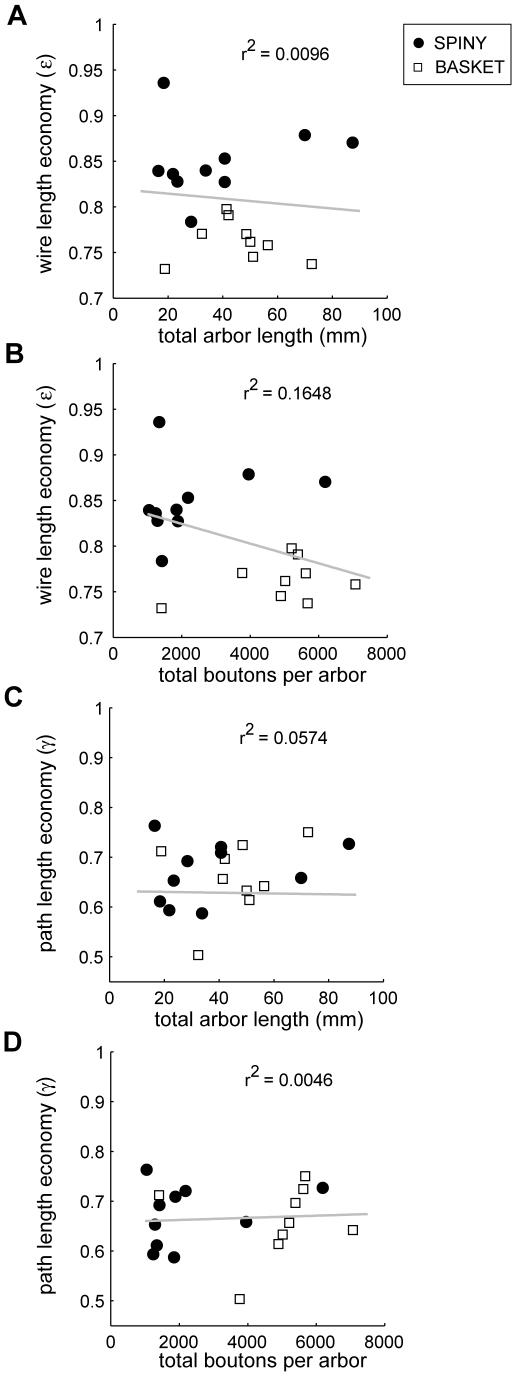
Wire length economy and path length economy uncorrelated with either arbor length or bouton number. (A) Wire length economy (ε) versus total axon arbor length (*L_AXON_*) (linear regression shown as solid grey line; slope  =  −0.000272 mm^−1^, intercept = 0.82, r^2^ = 0.0096), (B) Wire length economy (ε) versus total boutons per arbor (slope  = −1.08e−5 mm^−1^, intercept  = 0.85, r^2^ = 0.1648), (C) Path length economy (γ) versus total axon arbor length (*L_AXON_*) (slope = 0.0008 mm^−1^, intercept  = 0.63, r^2^ = 0.0574), and (D) Path length economy (γ) versus total boutons per arbor (slope  = 2.16e−6 mm^−1^, intercept  = 0.66, r^2^ = 0.0046) (n = 19). The lack of correlation implies they are scale-invariant economy measures.

To investigate wire length economy, we contrasted the total length of intracortical axon arbors to minimum-length graphs (Figures 3–7; see Table S1). Spiny cell axon arbors were not optimized for wire length (p<0.001, Wilcoxon signed rank test, one-sided; *ε*_spiny_ = 0.86±0.04, mean ± sd) with on average 5.66±2.93 mm excess wire per axon or 14±4% of total wire length (Figure 3). For example, a minimum-length graph connecting the same bouton set as a layer III pyramidal (spiny) axon arbor used 6 mm less wire or 15% of total axon length (Figure 4). Basket cell axons also were suboptimal for wire length (p<0.005, Wilcoxon signed rank test, one-sided; *ε*_basket_ = 0.76±0.02) and even significantly less economical than spiny cells (*ε*_spiny_ vs. *ε*_basket_: p<0.0005, Mann-Whitney U test, one-sided) (Figure 3) with on average 10.33±4.13 mm excess wire per axon or 24±2% total axon length. For instance, a minimum-length graph of a large layer III basket cell axon arbor used nearly 14 mm less wire or 24% of total axon length (Figure 5). In comparison, star graphs used around 40–50 times more wire than axons (*ε*_star_ = 0.02±0.01 and 0.02±0.02, respectively; see Figure 3). Both wire and path length economy measures were uncorrelated with either total arbor length or bouton number (Figure 6), suggesting they are scale-invariant measures and robust to incomplete axon arbor reconstruction.

**Figure 7:**
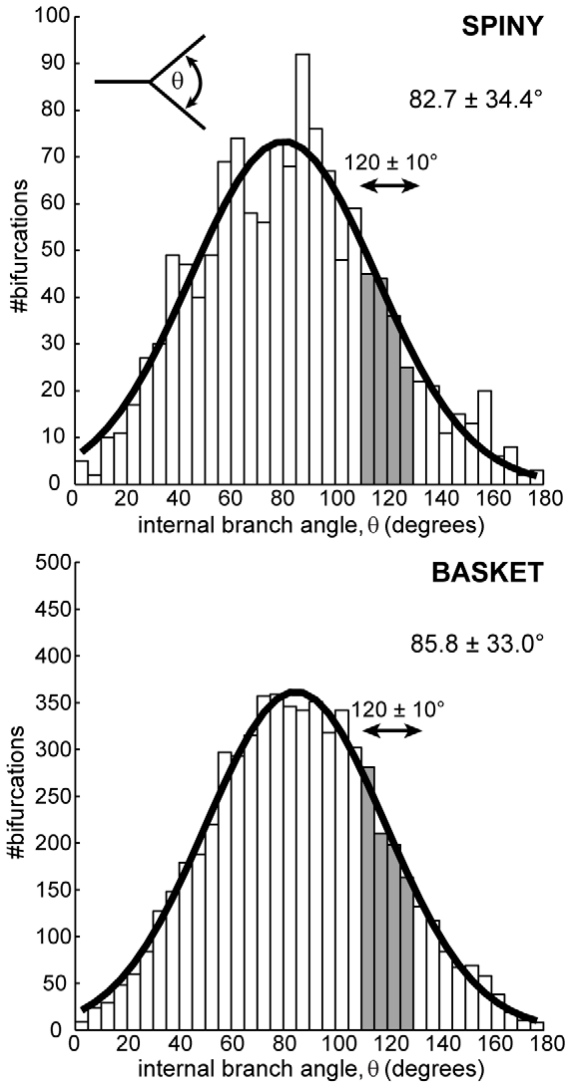
Axon branch points (bifurcations) are not generally Steiner points. Distribution of local internal (aperture) angles at neocortical axon arbor bifurcations did not match Steiner point angle condition of 120° (filled bars indicate ±10° range) for either spiny (*upper*, 12% within ±10° range from n = 1298 nodes) or basket cell classes (*lower*, 14% out of n = 6192 nodes). Inset (*upper*) shows schematically how internal branch angle measurements were made from axon arbor reconstructions. Best-fit Gaussian distributions are shown in thick black lines (spiny, μ = 80.3°, sd = 35.7°; basket, μ = 84.8°, sd = 34.5°).

Inserting additional vertices (akin to axon bifurcations) did not significantly reduce total arbor length. The ESMT algorithm inserted typically double or more (Steiner) points than actual axon bifurcations per arbor (spiny axons: 130±73 axon nodes vs. 621±518 Steiner points; basket: 632±348 axon nodes vs. 1662±528 Steiner points) but only marginally shortened total wire length (see Table S1). Rarely were these additional vertices co-located with actual axon bifurcation nodes (≤2.5 µm distance: spiny, 2.92±1.90% per arbor; basket, 8.45±4.62%). Here, axon internal (aperture) branching angles were distributed normally (spiny angle distribution, 82.7±34.4°, n = 1298 nodes; basket, 85.8±33.0°, n = 6192 nodes; see Figure 7). Regardless of algorithm, Steiner points require a 120° internal angle [13],[32],[33]. Yet few axon bifurcations met this condition (spiny, 12% and basket, 14% in range 120±10°; see shaded region, Figure 7). This discrepancy cannot be explained by, for example, local junction volume optimization [13] because while nearly three-quarters of all spiny axon diameter branching ratios were unambiguously of equal volume cost (74%, 965/1298) few of these matched the 120° prediction for equal volume cost (11%, 106/965). These results suggest the branching properties of intracortical axonal trees do not match those of wire-minimized Steiner minimal trees.

Crucially, if the purpose of axon bifurcations was to shorten arbor wire length (as predicted from the wire minimization principle) then supplying them as additional vertices for the MST algorithm (“MST nodes” results) should guarantee a wire-minimized arbor [31]. Yet in all cases this critical test resulted in longer not shorter arbors (spiny, +0.61±0.30 mm, p<0.005; basket, +2.46±1.23 mm, p<0.005, both Wilcoxon signed rank test, one-sided; see Figure 3), implying that the positioning of intracortical axon bifurcations is not consistent with shortening wire length.

Overall, these results, invariant to reconstruction completeness, suggest that individual excitatory and inhibitory intracortical axon arbors are not optimized for wire length and their branching behaviour does not match the predictions of the wire or local volume minimization principles.

### Origin of Excess Axonal Wire

**Figure 8:**
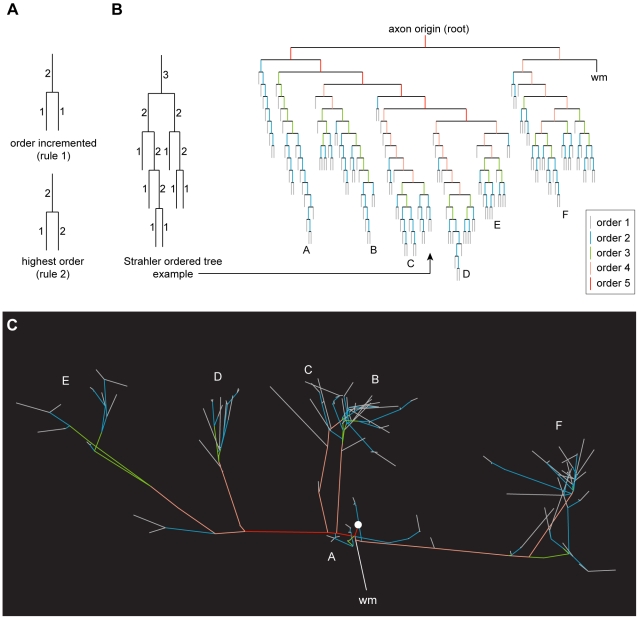
Topological ordering of an axonal tree. (A) Strahler ordering scheme maps axonal tree topology by applying two rules to increment the order of parent branch when its descendant branches have the same order (rule 1, *upper*) otherwise setting the order to the maximum order of children branches (rule 2, *lower*). Hence, in this centripetal ordering scheme terminal branches are labelled first-order and the root branch (axon origin) given highest branch order. Example application of order scheme to whole pyramidal cell axonal tree illustrated by (B) dendrogram (*right*), where each vertical line was colour-coded to represent branch order (see key) and black lines represents links except for section leading to white matter (wm), with a labelled subtree (*left*) showing application of numbering scheme, and (C) coronal view of axon graph representation (direct distances between morphological landmarks) with colour coded branches to match the dendrogram representation shown in (B).

To investigate potential sources of excess wire, we first used Strahler ordering [40]–[42] to characterise the branching structure of each axonal tree (for an example, see Figure 8). The Strahler ordering scheme has been widely used to quantify natural tree-like branching hierarchies including dendritic as well as axonal arbors [41],[42]. We chose this particular scheme to permit a direct comparison with previous work on the structure of intrinsic cortical axon arbors in cat visual cortex [41]. This centripetal ordering scheme gives a purely topological description of the axonal tree by labelling terminal branches as first-order (*k* = 1) and then incrementally ascending the tree hierarchy until reaching the root branch (axon origin), which has maximum order [42]. Here, spiny cell axonal trees had maximum order of 5 or 6 except for one arbor of 4, while most basket cells had maximum order 7 except for one of order 5 and one of 6 (c.f. 5–6 spiny & 5–7 smooth, [41]). In addition, for each arbor we classed internodal axon branches (*k*≥2) as either ‘*bouton-laden*’ (sections directly supporting one or more boutons) or ‘*bouton-free*’ (sections lacking any boutons).

One source of excess wire was the typically short (a few µm) distance between the last (most distal) bouton and the tapering tip of the axon branch, the terminal axon segment (see Figure S2). This source accounted for around 2% excess wire (spiny: 0.74±0.35 mm per arbor or 1.7±0.9% excess wire; basket: 1.28±0.31 mm per arbor or 2.3±0.6% excess wire). All subsequent analyses subtracted this source of excess wire.

**Figure 9:**
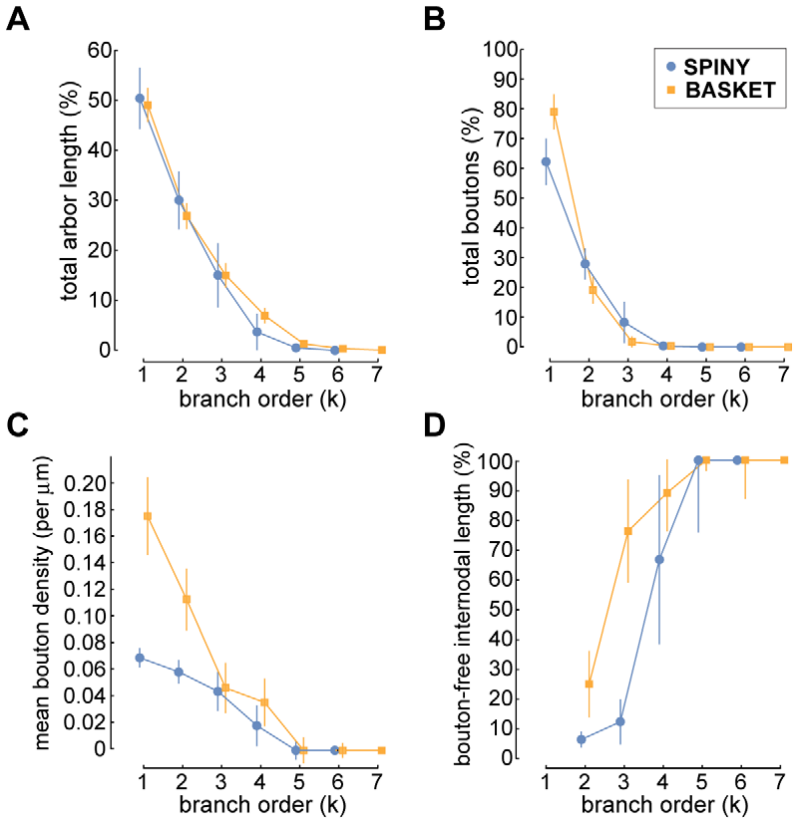
Axon length and number of boutons per branch order. For both basket and spiny cell axons, with increasing branch order there was a rapid decline in (A) percentage of total axon arbor length per arbor, (B) percentage of total boutons per arbor, and (C) mean bouton density (measured from ‘bouton-laden’ axonal sections only, so ignoring ‘bouton-free’ section length from the calculation – for distinction, see text), which was initially much higher from basket than spiny cell axons. Hence, the majority of axonal wire and boutons were found on first- and second-order branches. (D) Proportion of internodal axon length per branch order accounted for by ‘bouton-free’ sections increased with branch order with an offset between spiny and basket cell classes reaching 100% at fifth-order.

**Figure 10:**
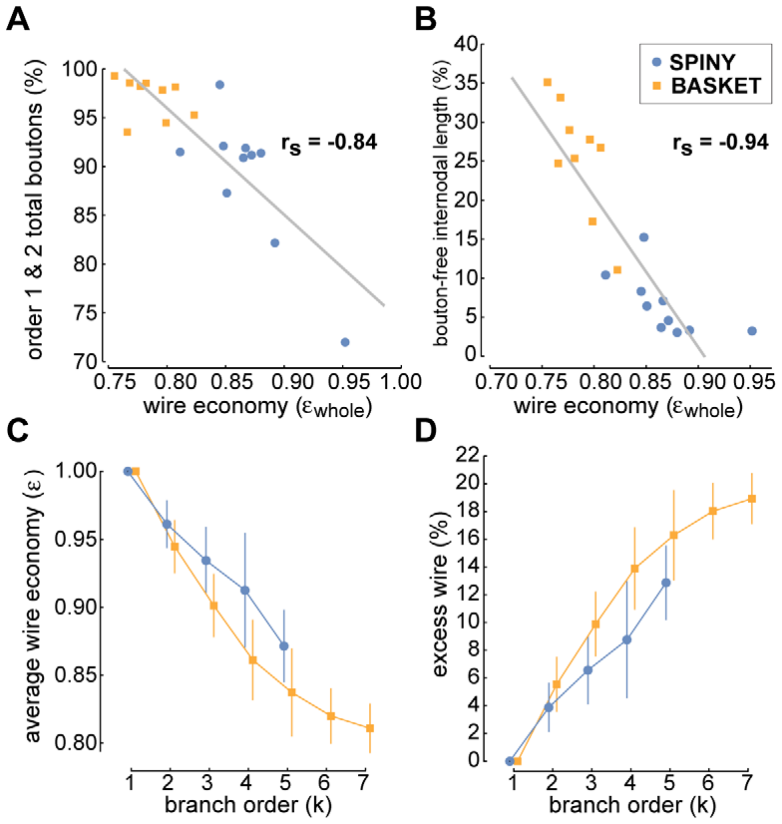
Excess axonal wire originates from nature of bouton distribution, ‘bouton-free’ internodal length, and branching complexity. (A) Whole arbor wire economy was negatively correlated with the proportion of boutons on first- and second-order branches (Spearman rank correlation, r_s_ = −0.84, p<10^−6^, one-sided; linear regression (solid grey line), slope  = −109.21, intercept  = 183.35). (B) Whole arbor wire economy was strongly negatively correlated with the proportion of internodal wire length due to ‘bouton-free’ axonal sections (Spearman rank correlation, r_s_ = −0.94, p<10^−6^, one-sided; linear regression, slope  = −1.93, intercept  = 1.75). (C) Average wire economy of axonal subtrees decreased with parent branch order towards whole arbor economy levels suggesting basket axon poorer wire economy was associated with their greater degree of branching complexity. (D) Percentage excess wire grew with branch order towards whole arbor levels implying each level of branching costs excess wire length in neocortical axons.

When examining how different wire-related arbor properties varied with branch order, we discovered that while the proportion of total axon length and bouton number, and bouton density all decreased with branch order, conversely, the proportion of internodal bouton-free axon length increased (see Figure 9). For example, first- and second-order branches accounted for the vast majority of boutons (spiny, 88.9±7.2% & basket, 97.1±2.1%; c.f. grouped 92±5% [43]) and axonal wire (spiny, 80.6±6.5% & basket, 76.1±3.7%; c.f. length uncorrected & grouped 82±6% [43]) (see Figure 9AB). In addition, mean bouton density (bouton-laden sections only) fell as branch order increased with, for example, basket cell first- and second-order branches having a greater density than spiny cells axons (e.g. at first-order: spiny, 0.07±0.01 & basket, 0.18±0.03 boutons per micron, or interbouton interval (ibi) 14.1 & 5.7 microns per bouton, respectively; c.f. grouped ibi 3–11 microns per bouton [43]), though thereafter bouton density declined similarly to zero by fifth-order (see Figure 9C). Importantly, we found whole arbor wire length economy was negatively correlated with the proportion of total boutons per arbor located on first- and second-order branches (Spearman rank correlation, r_s_ = −0.84, p<10^−6^, one-sided; linear regression, slope  = −109.21, intercept  = 183.35; see Figure 10A) suggesting wire length economy improved when boutons were more evenly spread over an arbor.

Internodal axon branches of basket cells are often myelinated and so lack boutons [8],[21] though this appears less prevalent in spiny cell axons [24],[44]. Here, we found internodal bouton-free length per arbor increased on average from a fraction of second-order total branch length (spiny, 5.5±2.7%; basket, 25.7±11.2%) to 100% by fifth-order (see Figure 9D). Whole arbor wire length economy was negatively correlated with the proportion of total axonal length that was bouton-free (Spearman rank correlation, r_s_ = −0.94, p<10^−6^, one-sided; linear regression, slope  = −1.93, intercept  = 1.75; see Figure 10B), indicating that arbors with a lower proportion of bouton-free internodal wire tended to be more economical. Recall ‘bouton-free’ wire length here refers to complete internodal sections lacking any boutons, which therefore might be myelinated, and does not count interbouton gaps on bouton-laden sections. Together these results suggest intracortical axon higher-order branches (k≥3) support fewer boutons per length and have proportionately more whole internodal sections devoid of boutons than lower-order branches.

To investigate the relationship between wire economy and axonal branching, we computed the wire economy of each subtree grouped by branch order (origin of parent branch became subtree root vertex) but excluding root branch (whole arbor). Recall wire economy was uncorrelated with bouton number or axon length (see Figure 6). Here, we found that as subtree branch order increased, starting from terminal branches (which after tip length correction were optimal) towards whole arbor, so the average subtree wire length economy progressively decreased (Figure 10C). Correspondingly, as subtree branching complexity increased so the proportion of excess wire increased asymptotically in increments of between 2–4% for spiny cell and 1–6% for basket cell axons (Figure 10D). Individual arbor rate of decline in wire economy between branch order levels was scaled by whole arbor economy level (spiny: r^2^ = 0.82, p<10^−6^; basket: r^2^ = 0.92, p<10^−6^). Thus, branching itself appears to cost wire, which may explain why basket cell axon arbors generally have poorer wire economy than spiny cell axons.

**Figure 11:**
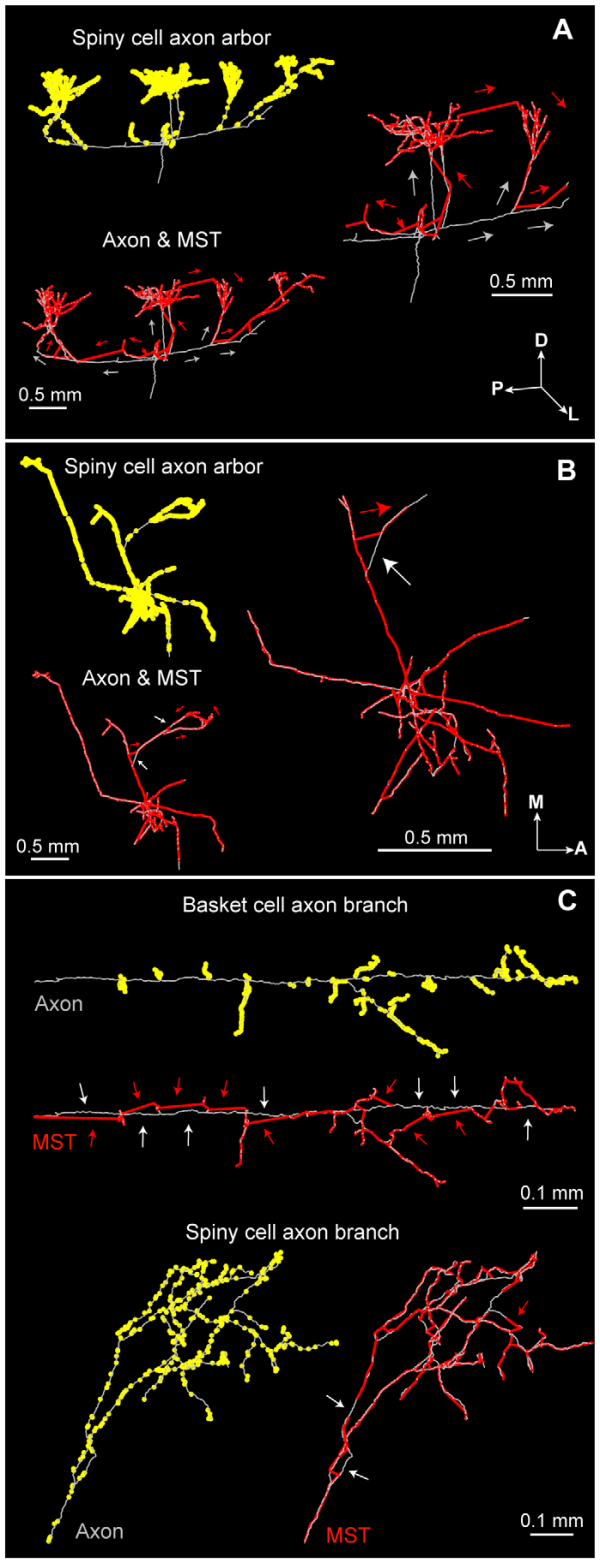
Examples of wiring ‘shortcuts’ by wire-minimization algorithms. (A) Low economy spiny axon arbor (*left upper*) wiring was significantly shortened by MST (*left lower*) shortcuts linking bouton-rich terminal patches while avoiding bouton-free primary and secondary axon collaterals. Magnified central region (*right*) shows numerous wiring differences in the flow direction from soma to tips between overlaid axon (grey arrows) and MST (red arrows) arbors. (B) High economy spiny cell axon arbor (*left upper*) wiring was only slightly shortened because MST (*left lower*) could find fewer shortcuts because of the more uniform bouton distribution over the sparsely branched axon arbor. Magnified central region axon and MST overlay (*right*) illustrates few differences in wiring pattern flow between these arbors. (C) Cell type-specific differences in wiring shortcuts: for a typical basket cell axons wiring shortcuts zig-zag between boutons ‘strings’ on terminal branches but avoid the main bouton-free axon collateral (*upper*), while for a typical spiny cell branch boutons are found on all orders of branching permitting very few shortcuts (*lower*). (Key: axon  =  grey lines, graph  =  red lines, boutons  =  yellow dots; anatomical axes: A, anterior; D, dorsal; L, lateral; M, medial; P, posterior).

Wire-minimization algorithms aim to shorten total wire length by simplifying a geometric problem without regard to any other objective function [32]. So it is understandable why the nature of the bouton distribution over an axonal tree, both in terms of local density and spaces of the arbor lacking any boutons (bouton-free sections), determines wire economy. A low economy spiny axon arbor, for example, directly links bouton-rich terminal patches instead of following the path of the actual but bouton-free main axon collateral, which runs tangentially to the cortical surface (see Figure 11A). Yet for the most economical spiny axon fewer shortcuts exist because boutons were more evenly spread over its arbor (Figure 11B). Particular to basket cell axon morphology, shortcuts ‘zig-zag’ between unmyelinated bouton-rich terminal branches avoiding myelinated bouton-free collaterals (Figure 11C, upper), a feature absent in spiny cell arbors (Figure 11C, lower). Moreover, in our sample the most economical axon had the lowest branch order (4), most boutons on its higher-order branches (28%), and nearly the least bouton-free wire (3.3% c.f. 3.1%). In contrast, the least economical arbor had the highest order (7), least boutons on its higher-order branches (1%), and most bouton-free wire (35%). The ESMT algorithm performed similarly in relation to the MST algorithm (see Steiner ratios in Table S1) suggesting economy was not related to algorithm performance. Together these results suggest that the origin of wire economy involves a combination of factors that constrain spatial (geometric) bouton distribution in particular the degree of branching complexity, the proportion of bouton-free internodal length, and the relative distribution of boutons over an arbor.

### Temporal Cost of Axon Arbors

In cerebral cortex, a low degree of temporal dispersion of synaptic input arrival times (standard deviation of latencies) is critical for the synchronization of distributed responses [45], rapid network oscillations [46], and coincidence detection within the millisecond range [47]. The degree of temporal precision is dependent on the anatomical and physiological characteristics of axonal wiring interconnecting cortical neurons. Hence, the minimum width of the postsynaptic temporal integration window is at least partly dependent upon the precision of intracortical architecture. An interconnected network of star trees, for example, would be expected to provide optimal temporal precision by (i) minimizing temporal dispersion, and (ii) preserving the distance-time relationship, so that signals from co-active neurons equally distant from a postsynaptic neuron they both innervate arrive simultaneously. In visual cortex, for example, these properties are believed to be important in promoting the temporal binding of spatially distinct co-linear visual stimuli [30].

**Figure 12:**
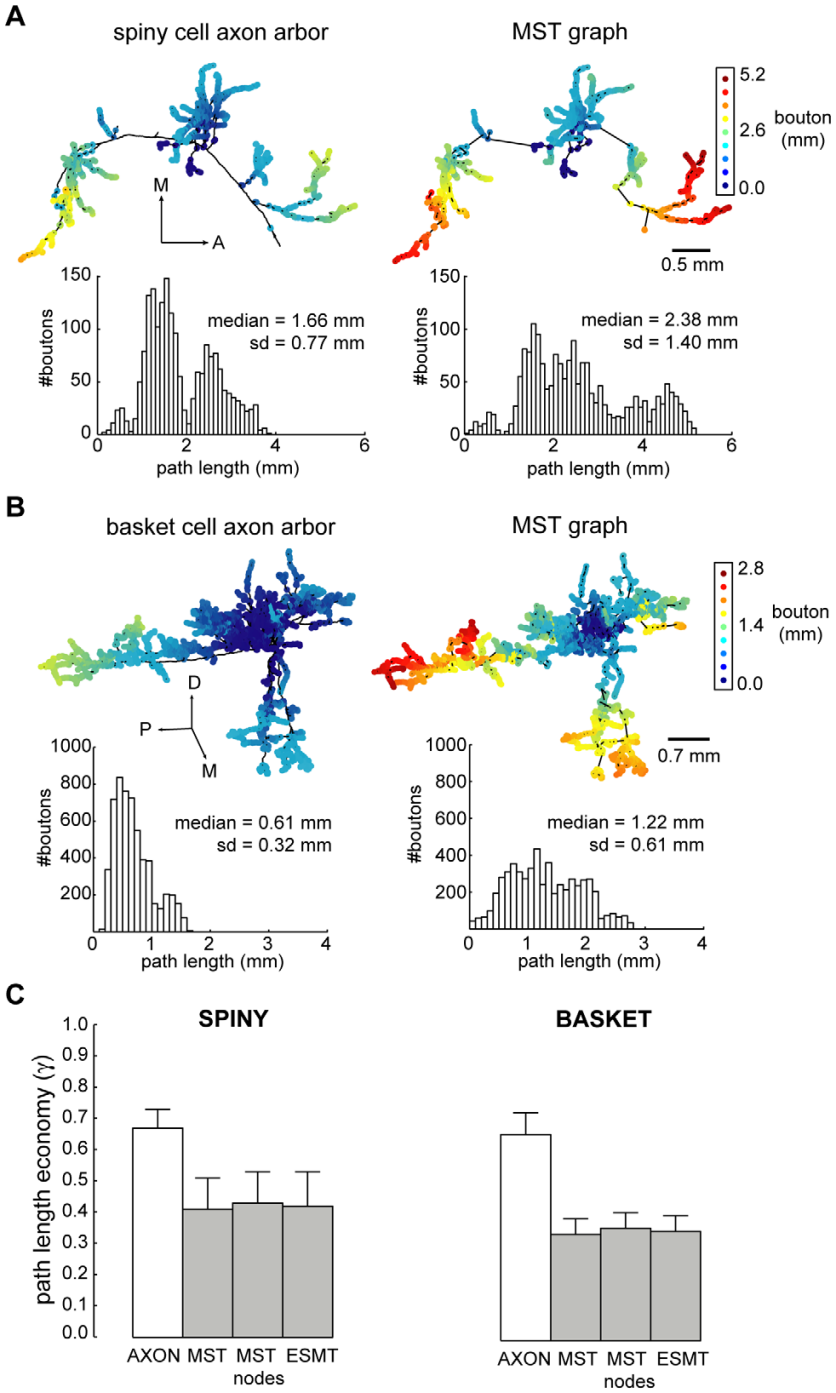
Path length economy of neocortical axons was suboptimal though superior to wire-minimized arbors. (A) Coronal view of example spiny cell axon arbor (*left*) and its MST (*right*): *upper*, shows wire length minimization generally increased path length from parent cell body along arbor to each bouton (dot colour codes for path length, see scale bar), and, *lower*, histograms show this results in a shift in path length distribution of these arbors from positively skewed to one more dispersed and symmetric. (Anatomical axes: A, anterior; D, dorsal; L, lateral; M, medial; P, posterior). (B) Surface view of example basket cell axon arbor (*left*) and its MST (*right*) shows, *upper*, a similar increase in path length (note different colour scale to (A)) compared with spiny axon arbor with wire minimization, and, *lower*, a spread in path length distribution. (C) Path length economy (γ) of spiny (*left*) and basket cells axons (*right*) was suboptimal (γ*_AXON_* = *P_STAR_*/*P_AXON_*, where *P_AXON_* is average path length from parent soma to each bouton in the arbor) though significantly greater than wire-minimization arbors regardless of whether or not (γ*_MST_* = *P_STAR_*/*P_MST_*) these inserted additional vertices (branch points) according to Steiner minimal tree criteria (γ*_ESMT_* = *P_STAR_*/*P_ESMT_*) or the actual axon bifurcations or nodes (γ*_MSTnodes_* = *P_STAR_*/*P_MSTnodes_*) were used.

Here, we investigated the temporal economy of axon arbors compared to wire-length minimized graphs (Figures 12–14; see Table S1), assuming a uniform conduction velocity at each part of the arbor. Example spiny and basket cell axon arbors demonstrate that wire length minimization increased both average path length (average axonal latency) and path length variance (temporal dispersion) (Figures 12AB). In general, average path length from axon origin (root vertex) to bouton for biological arbors (median ± sd, spiny, 1.13±0.41 mm; basket, 0.61±0.15 mm) was much shorter than wire-minimized MST (spiny, 2.04±1.30 mm; basket, 1.21±0.69 mm). Hence, spiny axon arbors were suboptimal for path length (p<0.005, Wilcoxon signed rank test, one-sided; γ_spiny_ = 0.67±0.06) as were basket cell axonal trees (p<0.005; γ_basket_ = 0.66±0.07). Yet axon average path length was significantly shorter than corresponding MSTs for both spiny (p<0.001, Wilcoxon signed rank test, one-sided; γ = 0.41±0.10) and basket cells (p<0.001; γ = 0.34±0.05) (Figure 12C). Path length variance of axon distributions was significantly less than MST distributions (spiny & basket, p<10^−6^, Brown-Forsythe modified Levene test). In contrast to wire economy, there was no difference in path economy between axon classes (γ_spiny_ vs. γ_basket_: p = 0.91, Mann-Whitney U test, two-sided) indicating that intracortical arbor temporal cost may be class-independent. Inserting additional vertices did not significantly improve path length economy with ESMT (spiny, p = 0.09 and basket, p = 0.47, Wilcoxon signed rank tests, two-sided; γ_spiny_ = 0.42±0.11, γ_basket_ = 0.35±0.05) though supplying axon nodes led to a small increase for MSTs (spiny, p = 0.17; basket, p = 0.07; both Δγ = +0.02) (see Figure 12C). Thus, with or without additional vertices and regardless of cell class, wire minimization yielded worse temporal economy than real axons.

**Figure 13 pcbi-1000711-g013:**
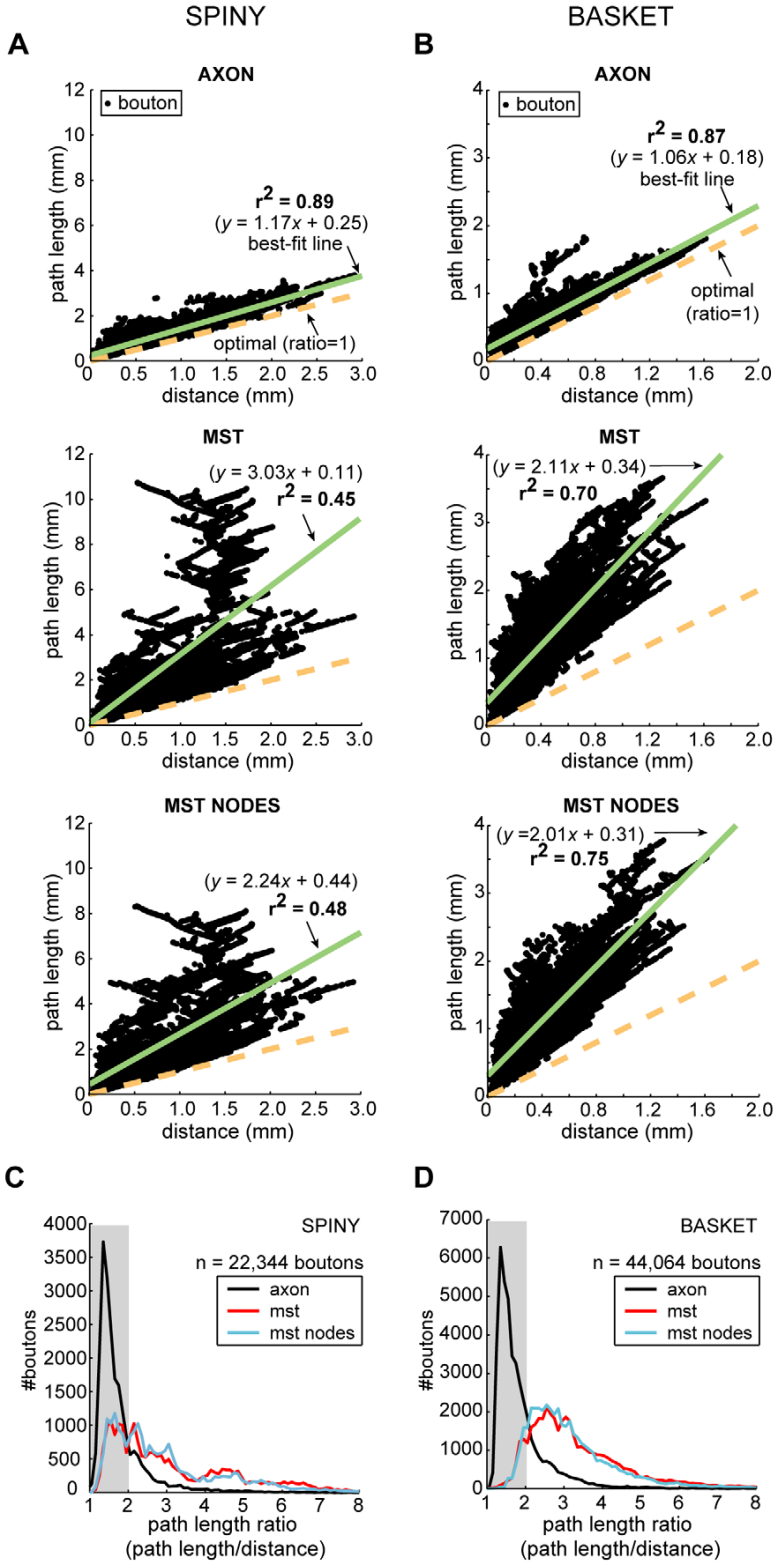
Neocortical axons, unlike wire-minimized arbors, preserve cortical distance-path length relationship. (A) Spiny cell axons (*top*) regression line (solid green line) diverged little from optimal slope (path length/distance ratio  = 1, dotted gold line) and was much better correlated compared with MSTs either without (*middle*) or with axon bifurcations (nodes) as additional vertices (*bottom*). Black dots represent single bouton measurements, *n* = 22,344 boutons. (B) Basket cell axons (*top*) regression line likewise diverged little from optimal slope and was marginally better correlated compared with MSTs either without (*middle*) or with the addition of axon bifurcations (*bottom*), *n* = 44,064 boutons. (C) Spiny cell axon path length ratio distribution (black line) showed a sharp initial peak followed by a slower exponential-like decay with 82% of ratio <2 (grey shaded region), compared with the broader distributions of MSTs without (red line) and with the addition of axon bifurcations (blue line) with 33–34% only of ratio <2. (D) Basket cell axon path length ratio distribution had a similar shape to spiny cells', 78% of paths with ratio <2, while the wider distributions of MSTs without and with axon bifurcations as additional vertices, with around 12–13% of ratio <2, peaked near ratio of 3. The strong similarity between spiny and basket axon path length ratio distributions implies a common (temporal) cost constraint mechanism.

**Figure 14:**
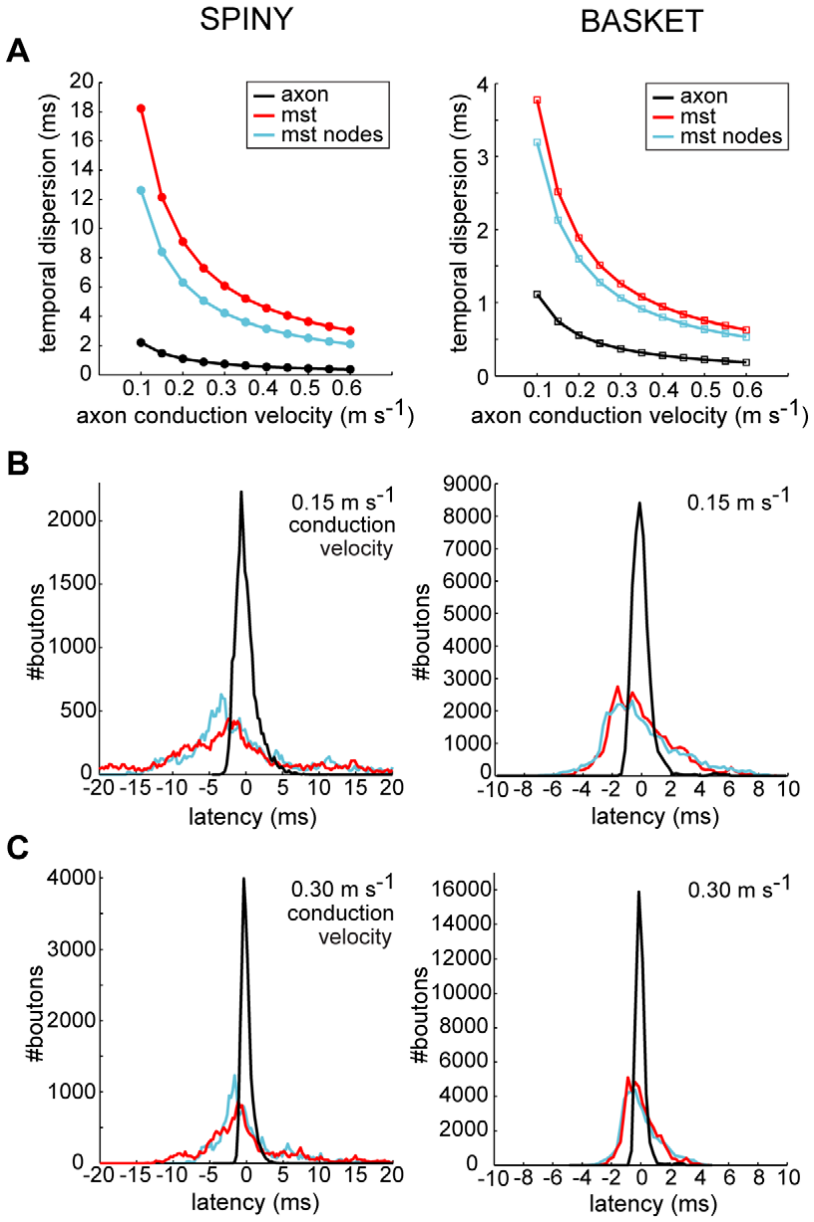
Temporal dispersion of neocortical axonal latencies was much less than wire-minimized arbors. (A) Degree of temporal dispersion (standard deviation) of spiny cell axons latencies (*left*, black line), independent of conduction velocity, was six to eight times and basket cell latencies (*right*, black line) around three times less than corresponding MST with (blue line) or without additional axon bifurcation points (red line), suggesting wire minimization increased temporal dispersion. Addition of axon bifurcations in MSTs reduced the degree of temporal dispersion. Standard deviation was measured by deviation from the respective regression lines shown for path length in Figure 13AB. Comparison of predicted latency distributions of axon arbors and MSTs at (B) 0.15 m s^−1^, and (C) 0.30 m s^−1^ conduction velocities illustrates the sharpness of axonal temporal dispersion compared with broader wire minimization results (N.B. total number of paths constant across conditions).

Figure 13 illustrates that for axons the relationship between cortical distance from axon origin to bouton and path length diverged only slightly from optimal (slope  =  path length ratio  = 1) albeit with a small offset (spiny, regression slope  = 1.17, intercept  = 0.25 mm, r^2^ = 0.89, see Figure 13A top; basket, slope  = 1.06, intercept  = 0.18 mm, r^2^ = 0.87, see Figure 13B top). In contrast, the path length of wire-minimized MST arbors, with or without axon bifurcations as additional vertices, diverged sharply from optimal with distance (spiny, slope  = 2.24–3.03, intercept  = 0.11–0.44, r^2^ = 0.45−0.48, see Figure 13A middle & bottom; basket, slope  = 2.01−2.11, intercept  = 0.31−0.34 mm, r^2^ = 0.70−0.75, see Figure 13B middle & bottom). Individual path lengths in axon arbors were typically less than twice the optimum length (82% spiny axonal boutons, n = 22,344 total boutons; 78% basket axonal boutons, n = 44,064 total boutons) while far fewer MST path lengths fell within this range (spiny MSTs, 33–34%; basket MSTs, 12–13%) (see shaded region in Figure 13CD). Axonal boutons with path length ratios of 2 or above were confined mostly to within 0.5 mm of parent cell body yet for MSTs such ratio values were found at nearly all distances. The similar, positively skewed shape of both spiny and basket cell axons path length ratio distributions (median ± sd, spiny  = 1.45±0.77; basket  = 1.53±0.86; see Figure 13CD) suggests high ratios were increasingly penalised compared with MST distributions which typically peaked later with a longer tail (spiny  = 2.44±1.77 with bifurcations & 2.57±1.96 without; basket  = 2.79±1.31 with & 2.94±1.58 without). These results suggest a common temporal cost mechanism may regulate intracortical axon morphology to preserve the distance-time relationship, which is especially important for the most distant connections within functional maps.

**Figure 15:**
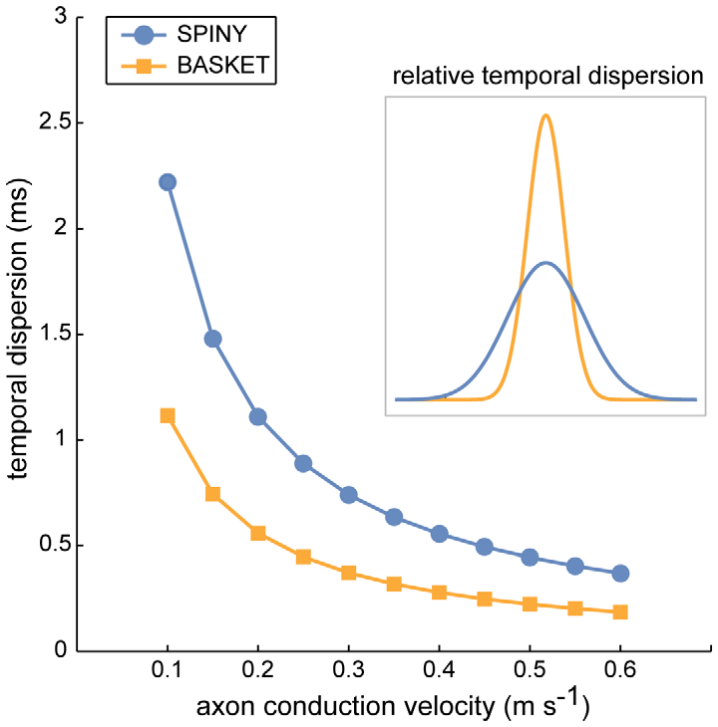
Temporal dispersion of basket cell axon latencies was approximately half that of spiny cell axons. Inset shows normalised Gaussian profiles of relative temporal dispersion independent of conduction velocity.

To predict the effect of wire minimization on temporal dispersion, we estimated axonal latency deviation about the regression lines (green lines shown in Figure 13AB) for both axon and MST data (Figures 14 and 15). Independent of conduction velocity, spiny cell axon temporal dispersion was 5.7 times less than MSTs with axonal bifurcation vertices and 8.2 times less than MSTs without axonal bifurcations (Figure 14A, left). For basket cell axon temporal dispersion, the corresponding values were 2.9 (with) and 3.4 (without bifurcation vertices) times less (Figure 14A, right). For instance, at 0.15 m s^−1^ conduction velocity latencies covered a narrower temporal window than MSTs (spiny, ±5 vs. ± >20 ms, Figure 14B left; basket, ±2 vs. ±8 ms, Figure 14B right), which was maintained when conduction velocity doubled to 0.30 m s^−1^ (spiny, ±2 vs. ±12 ms, Figure 14C left; basket, ±1 vs. ±4 ms, Figure 14C right). Figure 15 illustrates that the relative temporal dispersion of spiny cell arbors was double that shown by basket cell axons, which generally have greater branching complexity than spiny cell axons. Moreover, this difference is likely to be enhanced from the postsynaptic somatic targeting by largely myelinated basket cell axons [8],[21] compared to the postsynaptic dendritic targeting by mainly unmyelinated spiny cell axons [6],[17]. These results suggest the design of intracortical axonal arbors supports a low degree of temporal dispersion and a close relationship between distance and latency, prerequisites for intracortical synchronization [45], fast network oscillations [46], and coincidence detection [47], yet wire-minimized arbors (with or without branch points) demonstrate much poorer temporal precision making them ill-suited for these functions.

### Cost Trade-off for Axon Arbors

Based on these results, we hypothesized that *both* spatial and temporal costs simultaneously constrain intracortical axon arbors: empirical data here suggests that the least amount of wire was used to ensure that most axon path lengths were less than twice the minimum conduction delay. In classical network design problems, however, simultaneously minimizing both construction and routing costs is considered intractable because they are conflicting objective functions [9],[48]. Instead, approximation algorithms are used to find graphs representing a continuous trade-off between these two costs [9],[48].

To test this hypothesis and investigate the relationship between spatial and temporal arbor costs, to each axon arbor we applied the *light-approximate spanning tree* (LAST) algorithm [48], which *strictly* limits path length ratio through a single parameter, *α_LAST_*. Depending on *α_LAST_* value, the algorithm can generate at one extreme an MST (*α_LAST_*≫1) or at the other a shortest path tree (star tree) (*α_LAST_* = 1), with intermediate *α_LAST_* values generating hybrid MST-star graphs; for example, *α_LAST_* = 2 ensures that all path lengths are less than twice the minimum. To obtain a baseline for comparison, we generated for each axon 250 independently randomized trees spanning the same vertex set [49] and calculated their wire length and path length economy.

**Figure 16:**
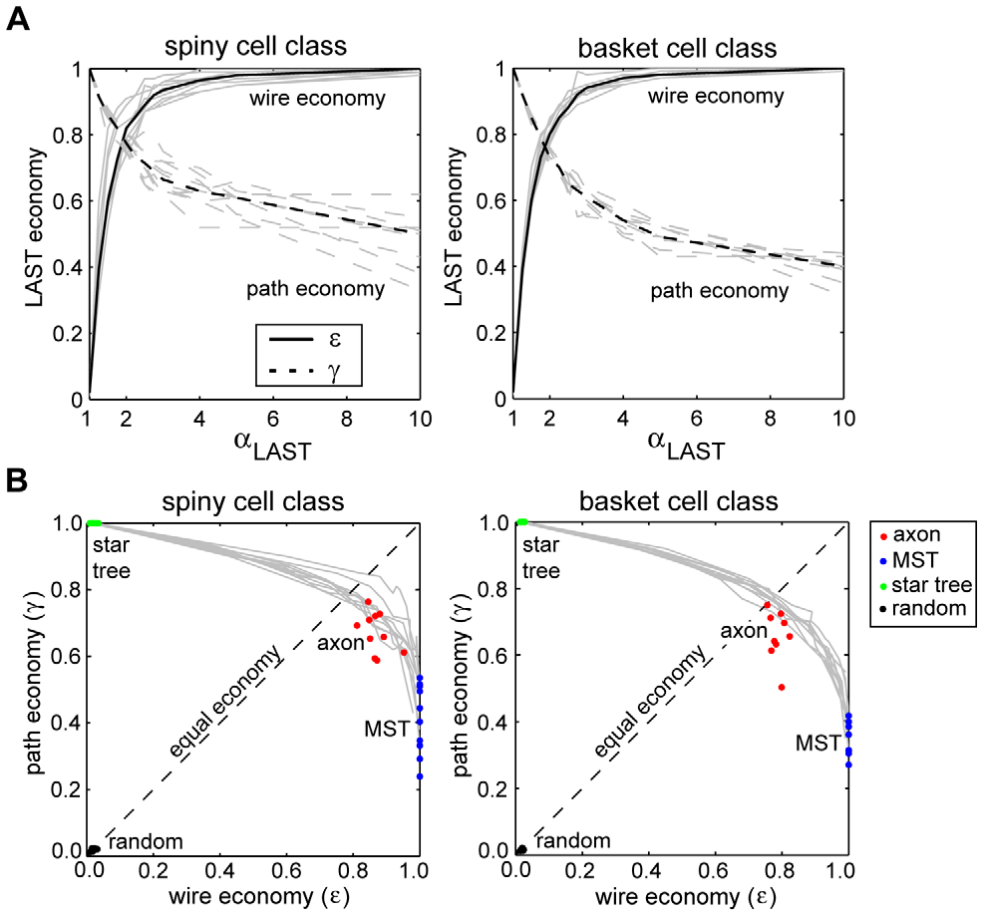
Neocortical axon arbor design represents a trade-off between spatial and temporal communication costs. (A) Light-approximate spanning tree (LAST) algorithm shows for each arbor that as the maximum path length ratio determined by *α_LAST_* increased so wire and path economy proved opposing objective functions for both spiny (*left*) and basket cell class axon arbors (*right*). At around *α_LAST_* = 1.9 the separate economy curves achieve equilibrium with parameters matched at ≈0.79. Light grey curves represent individual arbor results while thick black lines represent mean economy results (solid lines  =  wire economy, dashed lines  =  path economy). (B) Relative to trade-off curves in the economy plane generated by LAST algorithm (solid grey lines) the results show spiny (*left*) and basket cell axons (*right*) were suboptimal for wire length economy (*ε*) compared with wire-minimized MST and were suboptimal for path length economy (*γ*) compared with path-minimized star trees. Most neocortical arbors lay on or near the trade-off curves with a slight bias towards wire minimization relative to equal economy line (dotted black line). In comparison, randomized trees were close to the origin of the economy plane indicating the degree of axon economy.

For both spiny and basket cell populations, as *α_LAST_* increased so path economy decayed from unity to around half, while simultaneously wire economy rose rapidly from near zero to approach unity asymptotically (Figure 16A). The results support the hypothesis that wire and path length economies are generally opposing costs at least for this type of arbor. Around *α_LAST_* = 1.9 costs were balanced, ε = γ≈0.79 (Figure 16A). Combined in the ε–γ plane these curves created a continuous cost trade-off: commencing with star trees there was a gradual decline in γ with increasing ε until reaching the equilibrium point, where γ fell sharply down towards MST parameter values (Figure 16B). Hence, the trade-off gain in path economy becomes far more expensive in terms of wire cost to the left of equilibrium (Figure 16B). Importantly, the economy parameters of both basket and spiny cell class axons fall mostly on or around these trade-off curves (Figure 16B), suggesting that LAST algorithm offers a reasonable approximation to the underlying cost constraints on axon wiring. While a few axons were close to equilibrium, most had economy parameter values biased towards wire minimization. In comparison, randomized arbors gave simultaneously extremely poor both wire length economy (spiny, 0.017±0.006; basket, 0.014±0.004) and path length economy (spiny, 0.015±0.004; basket, 0.009±0.003) demonstrating the effectiveness of spatial and temporal cost optimization (Figure 16B). These results offer support for the hypothesis that neocortical axon arbor design represents a trade-off between spatial and temporal communication costs.

## Discussion

### Overview

For over a century, Ramón y Cajal's [10] conservation laws have guided research aimed at understanding the functional principles of neuronal morphology, a topic dominated recently by wire-length minimization of 2D arbors [12]–[16]. To our knowledge, this study is the first quantitative empirical test of Cajal's laws for whole 3D neocortical axon arbors within grey matter. Here, we discovered that neocortical axonal trees are not globally minimized for either wire length (material) or path length (conduction delay). Instead their three-dimensional branched design represents the trade-off of a modest amount of excess axonal wire (∼10–20% total arbor length, equivalent to roughly 3% extra grey matter volume [3]) to obtain a roughly two-fold gain in overall temporal economy and three-fold or more gain in temporal precision. In contrast, algorithms used here suggest wire length minimized arbors would significantly impair the temporal precision of neuronal network communication (Figures 12–14) whereas path length minimized arbors would demand at least an order of magnitude larger neocortex (Figure 3). Specifically, it appears axon bifurcations function to preserve the relationship between conduction time and cortical distance (Figure 13) and to tightly regulate the degree of temporal dispersion in transmission of axonal signals (Figures 14 and 15). From these axonal tree properties we infer that the highly interconnected intracortical network architecture, thought to underlie functional maps [7], [8], [21]–[23],[25],[30], is designed to be capable of operating with a high degree of temporal precision (e.g. for coincidence detection). In particular, inhibitory basket cell axon transmission seems capable of double the degree of temporal precision of excitatory spiny cell axon arbors (Figure 15), consistent with the notion that in cerebral cortex precise somatic inhibition sharpens coincidence detection of more broadly tuned excitatory signals [50]. Therefore, these results have implications for our understanding of neuronal communication and coding within cerebral cortex [1].

### Evaluation of Approach

The graph optimization algorithms were used here to measure the degree of optimality of single axons to investigate Cajal's laws of neuronal morphology and should not be considered as models of cortical circuit development (see ‘Developmental Considerations’). This type of approach is consistent with previous analyses of wiring economy [12], [14], [51]–[53] that have also relied on global information, mostly based on empirical data as done here. The rationale for wiring optimization is that it is the result of evolutionary pressure to maximize an organism's survival by selecting developmental mechanisms capable of generating the most efficient brain wiring [1],[12],[13],[16].

Minimizing total wire length and minimizing path length are distinctly different problems [9], [31]–[33],[48]. Although we cannot completely exclude the possibility that for a given vertex set other algorithms might find an arbor simultaneously optimal or very near optimal wire and path length economy (same connection topologies), we think in general it is unlikely because of the different objective functions and problem geometry. Consider, for example, a star tree where the optimal path length of a given vertex is a direct connection to the root vertex. To begin shortening the total wire length of this arbor requires that another vertex, whether fixed or inserted, be included in the path between root and the given vertex. Because of the triangular inequality of the Euclidean metric, a detour via this additional vertex will in general increase path length [31],[48]. So it follows that for any algorithm to further reduce total wire length implies that individual paths will become less direct and so longer. Therefore, we suggest the nature of the problem geometry will in general force any algorithm to trade-off wire and path length objective functions [9],[48], although other algorithms may achieve a better degree of trade-off. In any case such improved results would only serve to emphasize the suboptimality of cortical axon arbors.

The morphological and topological similarity of our sample with the only larger comparable studies of axonal trees [6],[41],[43] suggests it is representative of intracortical neurons in adult cat visual cortex. Since spiny and basket neuronal types are conserved [10],[11],[17] we would expect results here should generalise to other cortical areas across species, though it is possible the trade-off may vary according to functional requirements (e.g. enhanced temporal precision in auditory cortex). By labelling adult axon arbors in vivo we were able to analyse relatively stable, long-range axon arbor connectivity, which would not have been possible using axons obtained in vitro from neonatal brain slices (e.g. [20]). Our sample did not include any non-basket GABAergic cell types [17],[19],[20]. But we would expect to obtain comparable results from analysis of these missing cell types because they have similar properties to axons studied here such as total axon arbor length and internal axon branching angle [6],[43],[54].

Other known costs might affect the results. Metabolic cost, for instance, is generally considered a major resource limitation for brain organization and function [1]. Of the grey matter energy budget, signalling accounts for more than three-quarters with a smaller fraction ascribed to maintaining ionic equilibrium [55]. Because wire and path length costs should correspond with metabolic costs for ion channels and transporters at rest and when signalling, respectively, energy costs may be considered as implicit within the current approach. During development, material transport costs from soma towards the growing tips during axon extension [56], for example, are likely proportional to path length. Finally, for reasons of combinatorial complexity, we did not explicitly consider axon volume as a variable cost for optimization. Yet failure to find evidence supporting either whole arbor wire length or local junction volume minimization [13] here argues against whole arbor volume optimization. The current analysis, though not a complete description of all constraints, appears to represent a reasonable approximation to the main costs of neocortical axon arbor design.

To compare tortuous axon trajectories with straight graph edges, we measured the direct rather than actual distance between fixed points in the axon reconstructions (see Figure S1). It is reasonable to suppose that the difference in wire length between artificial and neuropil spaces would allow the graph extra length to avoid other neuronal processes and larger cellular obstacles such as capillaries [2],[3].

Dendritic processing also contributes to the signalling latency between presynaptic and postsynaptic cells [57]. Dendritic conduction delay is likely to be proportional to distance along dendritic branches from an axodendritic synapse to the cell body with conduction velocity dependent upon branch thickness and active and passive ionic currents [57]. Currently, it is impractical to trace in vivo each of the thousands of connections made by a whole axon arbor to their respective position on postsynaptic neurons, so anatomically-based estimates of dendritic delay must be based on knowledge obtained from previous work on individual cell pair tracings. Basket cell axons, for instance, invariably contact the cell bodies of postsynaptic neurons [8], [20]–[22] so it is likely that the temporal dispersion of basket cell axonal connections may not be significantly delayed by dendritic processing. In contrast, individual spiny cell axons mostly contact the proximal, medial or distal parts of the dendritic tree of other spiny neurons [3], [6], [23]–[28],[37], so here delay might be significant. However, there is evidence for a spatial segregation of synaptic inputs from different presynaptic sources on spiny cell dendritic trees [26], so it is possible that this delay might not greatly broaden temporal dispersion between two particular cell populations but simply provide an average timing offset between them, which may have a functional significance [58]. Thus, dendritic processing may increase the temporal dispersion of spiny cell signalling relative to basket cells (see Figure 15).

### Conservation Laws

Current results suggest that for neocortical axonal trees material conservation prevails over conduction delay conservation (see Figure 16). But here because of practical limitations we assumed a constant conduction velocity across the whole arbor (see Methods), which might underestimate temporal economy. For example, myelinated primary axon collaterals [34] could reduce latency to child branches without altering wire length, so shifting the trade-off closer to equilibrium. Recall many of the primary and secondary axon branches lacked any boutons (see Figure 9D) and so might be myelinated, which in the case of basket cell axons is most likely correct [8],[21],[22]. Moreover, there is evidence that evolution uses myelination to reduce conduction delay as wire length increases with brain size [59]. More accurate temporal costing might, therefore, reveal the two conservation laws are equally important.

Though the lack of wire optimization of single arbors here does not necessarily imply intracortical networks are suboptimal for wire length it does cast doubt on the applicability of the principle by itself to grey matter [12]–[16], especially given highly stereotyped connectivity patterns within neocortex [3], [6], [17], [19]–[23],[27]. Yet models claiming support for global wire minimization typically lack axonal branching and instead employ direct, parallel connections (star trees) between planar lattice points [14]. Hence, these models in fact optimize path length not wire length, which questions their validity to explain the organization of intracortical wiring functional maps in visual cortex according to wire length minimization only [14],[16]. Moreover, recent work suggests the only completely mapped nervous system (*C. elegans*) is not globally minimized for wire length ([51],[52] c.f. [53]). Independently, Kaiser and Hilgetag [52], using published gross connectivity matrices, recently reported non-optimal wire minimization in white matter between parallel pathways interconnecting visual cortical areas, which they too attributed to reducing communication delay. However, their study was concerned with unbranched axon bundles within white matter whose *mean* lengths were inferred not measured [52]. By contrast, here we traced and measured the length of the actual 3D trajectories of individual branched axon arbors within grey matter. If the results of Kaiser and Hilgetag [52] are later validated by empirical measurements of actual 3D individual axon lengths then this could imply the existence of a universal principle of cortical organization used both within (grey matter, this study) and between cortical areas (white matter) to optimize neuronal network communication.

While evidently correct for unbranched axons, the implication that axon arbor material conservation also leads to conduction delay minimization [11], [13]–[16] requires axonal branching to simultaneously save wire and path length, contrary to results from classical network design [9],[32],[48]. Isolated Steiner Y-junctions would appear to meet this requirement [13] provided the spatial arrangement of connections is compliant. Yet here the angle condition for Steiner junctions was rarely met by axon bifurcations (Figure 7). Moreover, linking together a set of individual Steiner junctions would not be expected to improve temporal economy because minimizing path length is not part of the objective function of Steiner minimal tree algorithms [32],[33], a point supported by ESMT results (see Figure 12C). Axon bifurcations in fact tend to worsen spatial economy (see Figures 3 and 10C) but improve temporal economy (see “MST nodes” results, Figure 12C). Basket cell axons, for instance, typically have a greater degree of branching complexity, less temporal dispersion but poorer wire economy than spiny cell axons. Indeed, if conserving wire length was the main determinant of axon morphology why do neocortical axons exceed third order branching when typically first and second-order axon branches account for virtually all boutons? Therefore, there is evidence from algorithms used here that axonal branching (increased parallelism) enhances temporal economy at the cost of spatial economy. Intriguingly, Ramon y Cajal [10] did note some examples of neuronal morphology “sacrificing economy of matter in favour of economy of time” (p. 105) though we suggest this is the general rule in grey matter. To optimize intracortical axon communication, we conclude that faced with a similar (neuronal) network design problem evolution has selected a trade-off where the spatial cost of arbor wiring is minimized subject to temporal cost limits.

### Developmental Considerations

Before considering how the developing axon arbor might be shaped by material and conduction delay conservation principles to attain its mature morphology, we need first to briefly outline the *in vivo* development of intracortical axonal trees and the different factors regulating axon morphology during cortical development.

Intracortical axonal trees examined here follow a characteristic pattern of development *in vivo* [60]–[72]. Spiny intracortical axons, for instance, begin with the main descending axon trunk emitting numerous collateral side branches that extend radially for up to a millimetre or so typically without branching (outgrowth phase) (for further details, see [60]–[68]). As these long primary collaterals gradually lengthen they then start to add distal branches but mostly interstitial secondary and tertiary branches at intervals along their length which then form crude clusters of collateral branches until reaching their maximum extent (elaboration phase). Finally, activity-dependent mechanisms are believed responsible for the increased branching frequency at some arbor locations and branch elimination at others to refine clusters (remodelling phase) [60], [61], [69]–[71]. Basket cell arbors similarly begin with the gradual extension of primary unbranched collaterals from the main axon shaft followed by the sprouting of distinctive interstitial side branches [72] though it is unclear whether or not these arbors are extensively remodelled. Thus, during cortical development both spiny and basket cell axon arbors increase in branching complexity.

Without tracking the development of individual whole cortical axons in vivo, it is not possible to directly determine how spatial and temporal communication costs might constrain local arbor growth. Yet evidently the initial structure of long unbranched axon collaterals radiating from the main axon trunk [60]–[61],[63],[66],[68],[72], similar to a star tree (see Figure 2A, right), implies that in the early stages of axon development minimizing conduction delay may take priority over material cost. The purpose of this initial radial outgrowth may be to rapidly cover the cortical space around the cell body to maximise potential connectivity and form a cortical scaffold with a precise distance-time relationship. Since the mature arbor appears more frugal with material cost this raises the possibility that during axon arbor development there may be a shift from temporal to spatial cost minimization. To investigate this idea, we need to understand how axon arbors develop.

During cortical development, the role of axon growth and branching is to find and synapse with numerous appropriate target neurons in order to construct a functional neuronal network. To find target neurons, the axon growth cone, the locomotory tip of the nascent axon, locally integrates multiple extracellular molecular signals via receptor activation to determine its direction and rate of outgrowth [56]. Extracellular ligands, which can trigger attractive or repulsive responses, include various growth factors (e.g. neurotrophins), short-range nondiffusible cell adhesion molecules (e.g. neural cell adhesion molecule, NCAM) and extracellular matrix molecules (ECM) (e.g. laminin), and long-range diffusible (e.g. netrins) and membrane bound concentration gradient cues (e.g. ephrins) [56]. At the growth cone, ligand bound receptors transiently increase the concentration of intracellular Ca^2+^ via influx through calcium permeable channels and/or release from internal stores [73],[74]. The frequency and spatial gradient of Ca^2+^ transients dynamically reorganize the growth cone's actin cytoskeleton (via calcium-dependent enzymes and Rho GTPases signalling pathways) to determine whether it extends, turns, retracts, splits (branch to create two growth cones), collapses, or pauses [74]. Axon morphology is determined by the organization of actin filaments, microtubules, and neurofilament cytoskeleton components [75]; though required later for axon calibre enlargement neurofilaments are not essential for axon elongation [76]. When moving slowly or paused, for example, the growth cone is enlarged with numerous sensing thin antenna-like processes (filopodia) that actively explore the local environment in a highly efficient manner [77] without affecting axon shaft orientation [78]. Yet when rapidly advancing, the growth cone is small and dome-like, lacking filopodia [73]. Mechanical tension generated by actin-related changes in the growth cone extend the axon in short straight sections between adhesive points [79],[80] with the rate of extension proportional to the degree of mechanical tension whether applied artificially by towing [81] or induced by extracellular signalling such as growth factors [82]. In a homogeneous growth medium lacking any guidance cues, a single axon through its intrinsic stiffness maintains an essentially straight course albeit with some oscillation [78], without growth-inducing extracellular signals no axon outgrowth occurs [83]. Individual growth cones can act independently [84], interact with others through long-range cAMP signalling [85], are modulated by global neuron state [86], and avoid contact with their own axonal processes [87]. Thus, a growing axon arbor can be described as performing a constrained parallel search of the developing neuropil guided by extracellular signals.

The overwhelming majority of cortical axon branches are interstitial rather than the result of growth cone splitting [88]–[90]. Delayed interstitial branching in cortical axons is strongly associated with earlier growth cone pausing behaviour [91],[92] while de novo interstitial cortical axon branching can be induced by local extracellular signals from diffusible chemoattractants like netrin-1 and a range of growth factors [91]–[94]. Interstitial branches are formed following Ca^2+^ transients that locally disrupt the actin cytoskeleton to reorganize actin filament and microtubule arrays [92],[95]. Other extracellular molecules such as diffusible chemorepellent Sema3A can, however, stop collateral branching by inhibiting growth cone pausing [93]. Hence, calcium signalling is implicated in axon extension, branching, and turning though these responses can be modified or reversed downstream in the signalling pathways [73],[74]. The initial stages of cortical axon development do not appear to depend on electrical activity [96],[97],[98] but on Ca^2+^ transients [73],[92],[94], which can also be triggered by electrical activity. For instance, Ca^2+^ transients originating from intracellular stores induced by either strong depolarization or receptor activated signals such as by growth factor ligands regulate neurotrophin secretion [99]. Later cortical axon remodelling does, however, depend on patterned electrical activity [61],[96],[98],[100]. Thus, intracellular calcium signalling appears central to controlling axon arbor development.

Growth factors are necessary for neuronal outgrowth, differentiation, and survival (see [101]). Multiple growth factors contribute to neuronal development including the neurotrophic family of molecules structurally related to nerve growth factor (NGF) such as brain-derived neurotrophic factor (BDNF) and structurally unrelated growth factors such as basic fibroblast growth factor (FGF-2) [101]. A target-derived growth factor signal travels from the distal axon to the cell body via both slow retrograde axonal transport of internalised ligand bound receptor complex endosomes and faster direct signalling cascades [102]. At the cell body, these signals through gene expression control the synthesis of neuronal proteins required for growth and inhibit programmed cell death [101]. Neuronal survival does not generally depend on a single growth factor and dependency can switch according to developmentally regulated changes in ligand availability, receptor expression or pathway response [101],[103]. In addition, specific growth factors can produce differential effects on axon outgrowth rate and branching probability for the same neuronal type [101], [104]–[108]. Importantly, axon branches receiving and supplying growth factor to the cell body survive along with the cell body itself but those that do not wither [109], suggesting growth factors are capable of selectively maintaining those axon branches important to neuronal survival. Therefore, extracellular growth factor molecules can selectively regulate axon arbor morphology.

Competition for resource-limited growth factors could explain how material and delay conservation principles drive or at least influence intracortical axon arbor development. To compete with other cortical neurons for survival, axons must rapidly obtain growth factors from target sources and then transport their signals back to the cell body as quickly as possible. To obtain growth factors rapidly, the existing axon must extend directly towards a source, an imperative that might drive material conservation. For example, calcium-dependent de novo axon branch induction by a variety of growth factors including FGF-2 is directed towards a localised source [106], and the incremental extension of an axon branch directly between discrete sources of growth factor [106],[110] produces morphology similar to bouton strings observed on cortical axon branches. Over longer distances and even in complicated spatial environments, pathfinding using local chemical gradients or contact based cues can yield the shortest trajectory to target sources [111],[112]. Furthermore, growth cones may be optimal at sensing growth factor concentration gradients in vitro [113]. To quickly transport growth factor signals from distal axon to cell body, an axon branch gains a competitive advantage over all others if the retrograde axonal transportation delay is shorter than for other axon branches regardless of whether they derive from the same (intra-axonal competition) or a different neuron (inter-axonal competition). Competition for the shortest transportation delay for growth factor signals to the cell body might drive conduction delay conservation and resource limitation would lead to pruning branches with longer delays. Taken together, these forces naturally lead to a trade-off between axon extension directly towards a growth factor source and retrograde signalling delay because while, for example, the axon of one neuron may extend a shorter branch to a target source than another, it will only gain a competitive advantage if the overall transportation distance is shorter.

The stages of intracortical axon development may be explained within this framework by the differential effect of multiple growth factors acting on the same growing axon. For example, secreted insulin-like growth factor 1 (IGF-1) facilitates neuronal survival and axon outgrowth of unbranched corticospinal tract axons towards distant targets while BDNF promotes their branching and arborization but not outgrowth [114], suggesting that multiple growth factors may act in concert on the same extrinsic axon to coordinate the different phases of arbor formation. It is possible that intrinsic axons develop in a similar manner but not necessarily using these particular growth factors in the same roles, even though both are expressed in neocortex postnatally [115],[116]. One type of growth factor (or combination of growth factors) might, for instance, support the initial rapid extension of long unbranched primary axon collateral to create the ‘spokes’ for a rapid transport system for growth factor signalling. Next, the dominance of another type of growth factor might then promote greater branching in the elaboration phase. Provided subsequent axon additions do not curve back towards the cell body, new connections formed by these secondary and tertiary branches will inherit a low path length ratio (see Figure 13CD). Finally, in the remodelling phase, homeostatic regulation may, as suggested for cortical dendritic arbors [117], maintain a total cost budget (derived from resource limits of available growth factor(s)) so that expansion in one part of the arbor may result in pruning elsewhere in the same arbor. Indeed, evidence exists for a push-pull branching mechanism during cortical axon arbor development based on the relative difference in local Ca^2+^ transient frequencies between branches [118]. Thus, within this competitive framework the stages of in vivo intracortical axon formation might be explained by developmentally regulated phases in neuronal dependency on multiple growth factors.

During its growth a cortical axon will encounter obstacles in the neuropil including others axons, dendrites, glia, and blood vessels [2],[3] and extracellular signals [56], both of which may constrain its trajectory. Current evidence suggests that dendritic tree (e.g. [119]) and astrocyte and oligodendrocyte glial cell maturation lags behind axonal development [120],[121] while capillary blood vessels, typically 2–3 µm diameter during early postnatal development, co-develop with intrinsic axons through common molecular guidance cues [122]–[124]. These observations suggest that the majority of neuropil obstacles may either be arranged to suit axonal tree development or avoided by small deviations in axon trajectory including axon-axon encounters. Recall before our analysis here we took into account axon trajectory deviations (see Materials & Methods). In any case, according to the growth factor mechanism proposed above, any large obstacles leading to grand excursions of axon length between neurons during development would typically be eliminated through growth factor competition and so would not appear in the adult arbors analysed here. Similarly, a neuron whose axon arbor becomes too restricted by local neuropil inhomogeneity might not survive into adulthood because of insufficient growth factor. Extracellular signals limiting axon growth patterns may also provide anatomical constraints on arbor economy [56]. Recent work in both visual and barrel cortex suggests, however, that inappropriate branches formed by initially exuberant arbors are later eliminated to produce the precise laminar or topographic specificity of mature intracortical arbors [68],[125], suggesting that arbors might, at least in some cases, be established first according to economy principles and later pruned according to the expression of laminar or spatial delimiting cues (e.g. [126]) without greatly affecting arbor economy. Regardless of whether growth cones might be optimal at finding target sources in vivo (see [113]), it is unknown whether, subject to anatomical constraints above, the competitive mechanism proposed here for the regulation of branch extension and pruning is optimal or not. It is difficult to test this because we know of no existing algorithm guaranteed to find the global optimum for this type of dynamical system problem. In addition, there are no published quantitative data for any species concerning the amount of intrinsic axonal wire used over the course of cortical development for comparison with the proposed mechanism. Therefore, at present the most we can conclude is that we would expect axon arbor development based on growth factor competition to be highly efficient.

How might the growth factor mechanism be affected by histological differences between cortical areas [127]? Cortical regional specification is itself controlled by extracellular signalling patterns [128],[129]. If area-specific histological differences occur postnatally during intrinsic axon outgrowth, such as in barrel formation in rodent somatosensory cortex [130], then growth factor competition will most likely ensure that the neurons with the most economical axons are retained while others are lost independently of changes in the composition of the neuropil. Otherwise cortical specification signals might compensate for differences in neuropil composition by altering the pattern and/or level of multiple growth factor signals to regulate the phases of axon development, e.g. increased neuronal density may require an increased levels of available growth factor [101].

In summary, we have proposed a potential mechanism based on growth factor competition to explain how and why material and conduction delay conservation principles might shape the development of intracortical axon arbors independent of cortical region. This proposal could be tested by tracking and manipulating the development of single intrinsic cortical axon arbors in vivo (e.g. [90]) to discover whether competition between axonal processes and subsequent pruning is related to these two conservation principles in the manner described.

## Materials and Methods

### Ethics Statement

All surgical procedures followed the German Welfare Act and were in accordance with European Communities Council Directive 86/809/EEC.

### Anatomical Labelling

Nine adult cats (8–14 months old) underwent anatomical labelling experiments. The surgical and anatomical labelling methods used here have been reported previously [21]–[23]. Briefly, anaesthetised, paralysed animals were used for intrinsic signal optical imaging and subsequent labelling of visual cortical neurons using intracellular and bulk injection of biocytin or biotinylated dextran-amin (ABC, Vector Laboratories, Burlingame, CA, USA). After completion of the in vivo recordings and injections the animals were perfused with a fixative and tissue blocks of region of interest were sectioned on vibratome. The labelling was revealed with the avidin-biotin complexed horseradish peroxidase technique [131] and the sections were dehydrated and embedded in resin on slides. The entire axonal and dendritic trees of well-labelled cells were reconstructed in 3D at ×1000 magnification using the computer-aided Neurolucida reconstruction system (MicroBrightField, Colchester, VT, USA).

### Analysis

#### Axon graphs

The topology of a single axon's three-dimensional connectivity may be represented by a rooted undirected weighted tree, *T* = (*V*,*E*,*c*), where *V* is the vertex set, *E* is the edge set connecting vertex pairs, *e* = (*u*,*v*), and *c* is a nonnegative weight or cost associated with each edge *c*(*e*) = *c*(*u*,*v*), where (*u*,*v*) 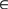 *T* [31]. The vertex set of an axon was composed of *N* axonal presynaptic en passant or terminaux bouton (presumptive synaptic connection) plus a single root vertex, *r*, representing the axon origin, |*V*| = *N*+1. These are referred to “fixed” vertices because their position was not altered. A vertex was defined by its location in three-dimensional Euclidean space *V* = (*v_x_*, *v_y_*, *v_z_*) relative to axon origin, *r* = (0, 0, 0). Each bouton was assumed to make at least one synapse [3]. To directly compare axons with graphs, we measured the direct rather than actual curvilinear distance between morphological landmarks (cell body, axonal bifurcations, axonal boutons, and axonal endings) with edge cost proportional to axon length (see Figure S1). This approach made it unnecessary to correct for the lengthening effects of axon tortuosity caused by geometric hindrance of other cellular elements such as cell bodies, glia, and blood vessels present in the neuropil [2],[3]. For spiny cell axons only, we excluded from the total axon length the final segment of myelinated axon descending into the white matter, which forms no connections in the cortical cylinder of the parent soma-dendrites (see Table S1). We applied Strahler's centripetal ordering scheme [40],[42] to characterise the topology of axonal trees (see example shown in Figure 8). The order (*k*) of each branch *i* (which in the graph representation may consist of a sequence of one or more edges terminated at axon bifurcation or ending) began at terminal branches, which were labelled first-order, *k_i_* = 1. Non-terminal axon branches then received a number based on the order of their child (descendant) branches, *k_j_*. If all child branches had the same order then the parent branch *k_i_* = max(*k_j_*)+1 otherwise *k_i_* = max(*k_j_*). The root branch (starting at axon origin) took the largest order often called the ‘Strahler number’ of the tree. Note because we occasionally encountered three-way axonal junctions the axon graphs here were not always strict binary trees. Unless otherwise stated all graph optimization analysis was performed using MatLab™ (Mathworks Inc, Natick, MA, USA).

#### Wire length analysis

We used Prim's minimum spanning tree (MST) algorithm [31] which is guaranteed to find in polynomial time a tree *T* spanning all fixed vertices of graph *G* such that the total cost is the minimum among all possible spanning trees of *G*, min *c*(*T*) = 
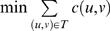
. Here, *G* was a complete graph with all distances measured. In addition, to test whether actual axon branch nodal points shorten total wire length, we applied the MST algorithm to a vertex set containing fixed vertices supplemented with the set of axon bifurcation locations *X* from the same arbor – referred to as “MST nodes” results. A shorter Euclidean Steiner minimal tree (ESMT) may exist if an additional set of Steiner points *S* (whose locations are determined by the algorithm to minimize the objective function to shorten total length) are inserted such that *Z* = *V* 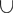 *S* [33]. *Z* is the combined set of fixed vertices (presumptive synapses and axon origin) and Steiner points. But the ESMT problem is known to be at least *NP*-hard for all dimensions *d*≥2 [32]. We applied the only ESMT heuristic algorithm currently available for large (>500) multi-dimensional vertex sets [33] (http://www.tapmi.edu.in/download/heuristic.zip). However, heuristics are not guaranteed to find global optima [32]. Thus, for a given vertex set, an MST gives the upper bounds and ESMT the lower bounds of total cost minimum [33]. The difference between MST and ESMT algorithms is that the MST algorithm cannot insert any additional vertices (see Figure 2B). The minimum length of a subtree was found by applying MST algorithm to a vertex set formed of fixed vertices (boutons) of the parent and all its child branches with the starting position of the parent branch as root vertex of the subtree. Subtree wire economy was calculated by dividing subtree minimum length by corresponding axon subtree length (excluding last bouton to tip length).

#### Temporal cost analysis

Temporal cost was estimated using the path length from axon origin to each bouton of an arbor. A path *p* is defined as a non-repeating sequence of vertices connecting vertex *u* with vertex *v* in *T* [31], *p* = <*u*, …, *v*>. Here, path length was defined as the sum of edge costs in *p*, *d_T_*(*u,v*) = 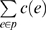, where *p* was the shortest path, the path of least cost among all possible paths in *T* between vertex *u* and vertex *v* found using Dijkstra's algorithm [31]. By definition, for all ESMT, LAST, and MST there exists a path connecting every pair of vertices in *T* [31]. To estimate latency in either axon or artificial arbors, we divided individual path length by a uniform conduction velocity over all edges of rooted tree, so all edges were assumed to have homogeneous conduction properties such as constant diameter, ion channel densities, etc; no numerical simulations of action potential propagation were performed. The assumption of a uniform conduction was necessary to allow a fair comparison between artificial and real axon arbors because, for reasons of combinatorial complexity, graph edges lacked a thickness dimension.

#### Branch node analysis

At each branch node, we computed the internal branch angle between adjacent axon segment vectors forming a plane with the node. In accordance with the definition of ‘local’ [13], we obtained from the reconstruction data the diameters of trunk and each adjacent branching segment within 1–10 microns of an axon bifurcation. The diameter of an individual trunk or branch was considered ‘unambiguous’ if diameter did not vary within this ‘local’ region. Local volume cost per unit length was calculated from cross-sectional area of trunk or branch by assuming a circular axon profile [13].

#### Trade-off analysis

To investigate the relationship between path and wire length optimization, we applied the light approximate spanning tree (LAST) algorithm [48] to the bouton distribution of individual axon arbors. This algorithm performs a depth-first traversal of the minimal spanning tree adding a shorter path when the existing path length ratio, the ratio of actual versus minimum path length, for any given vertex exceeds the path length ratio limit set by parameter *α_LAST_*. This algorithm inserts no additional vertices. Hence, the LAST algorithm ensures that the path length ratio of all vertices fall within the range [1, *α_LAST_*).

#### Randomized arbors

To provide a baseline for comparison with optimal and biological arbors, for each arbor we generated and measured the total wire length and average path length of 250 independently randomized spanning trees [49]. Each randomized spanning tree was generated from the fixed vertex set of boutons and axon origin (root vertex) of the real arbor using Wilson's algorithm [49]. Briefly, this algorithm begins with the current tree, consisting of the root vertex, and then performs a loop-erased random walk between the remaining vertices. When the random walk visits a vertex belonging to the current tree it is terminated and the path formed by the random walk is added to the current tree. The algorithm continues until all vertices are spanned [49].

### Statistical Analyses

All statistical analyses were performed with aid of R statistical package [132]. Statistical tests of significance for pairwise comparisons were performed with Wilcoxon signed rank test and for unrelated design with Mann-Whitney U test with 1% significance level.

## Supporting Information

Table S1 Individual axon arbor results. (A) Spiny cell axon wire length economy. (B) Basket cell axon wire economy. (C) Spiny cell axon path length economy. (D) Basket cell axon path length economy.
(0.08 MB PDF)

Figure S1 Original axon arbor reconstruction data converted into axon graph. (A) Schematic illustration of converting part of an axon arbor reconstruction (*left*) showing tortuous path of axon between boutons (e.g. light blue line) into (*right*) a graphical representation where direct instead of actual distance between boutons (i.e. compare lengths of light blue lines) was used as edge length (wire cost) to allow ready comparison with artificial arbors minimized for wire and/or path length. (B) Example of conversion process for whole spiny cell axon arbor (shown in Figure 4A) from original reconstruction (*left*) to axon graph representation (*right*), where branches without any boutons are classed as ‘bouton-free’ sections (orange line) and those with one or more boutons are classed as ‘bouton-laden’ sections (dark blue line).
(1.13 MB EPS)

Figure S2 Tapering tip correction explained. Schematic diagram showing distance between last presynaptic bouton (filled yellow circle) and tapering tip of axon branch represents a source of excess axonal wire not included in the analysis. Axonal tapering may provide improved passive signal propagation to the last bouton.
(0.25 MB EPS)
