# Supplementary material for: Neocortical Axon Arbors Trade-off Material and Conduction Delay Conservation
Source: PLoS Comput Biol. 2010 Mar 12;6(3):e1000711. doi: 10.1371/journal.pcbi.1000711 (PMC2837396; doi:10.1371/journal.pcbi.1000711)
Supplement: Table S1 — Individual axon arbor results. (A) Spiny cell axon wire length economy. (B) Basket cell axon wire economy. (C) Spiny cell axon path length economy. (D) Basket cell axon path length economy. (0.08 MB PDF) [file pcbi.1000711.s001.pdf]

TABLE S1. Individual Axon Arbor Results

TABLE S1 (a) SPINY AXON WIRE ECONOMY

axon	*cell type*	*boutons*	*Original axon arbor total length(mm)*	*Axon Graph total arbor length(mm)* L_t	*MST total arbor length (mm)* L_{MST}	*Wire Economy ratio (axon)* $\varepsilon = L_{MST}/L_t$	*Excess wire (mm)* $(L_t - L_{MST})$	*ESMT/MST Steiner Ratio* $\rho_3 = L_{ESMT}/L_{MST}$
oi15rpy4	pyramidal	1879	45.82	40.01	33.11	0.83	6.90	0.991342
oi24rpy1	pyramidal	1046	18.57	16.47	13.83	0.84	2.64	0.995567
oi27rpy1	pyramidal	3949	85.58	68.69	60.34	0.88	8.35	0.993523
oi27rpy2	pyramidal	2183	47.37	40.28	34.36	0.85	5.92	0.992565
oi28rpy1	pyramidal	6184	109.74	87.32	76.01	0.87	11.31	0.993033
oi33lpy1	spiny stellate	1348	23.85	21.76	18.19	0.84	3.57	0.991800
oi53rpy1	pyramidal	1232	19.59	18.43	17.25	0.94	1.18	0.994059
oi57rpy1	pyramidal	1844	36.91	33.80	28.40	0.84	5.40	0.991882
oi57rpy2	pyramidal	1295	22.99	23.14	19.16	0.83	3.98	0.994851
oi57lpy6	pyramidal	1429	29.78	28.41	22.27	0.78	6.14	0.992700
mean	-	2239	44.02	40.85	35.19	0.86	5.66	0.99
SD	-	1618	30.56	23.15	20.43	0.04	2.93	

‡ includes subtraction for length of non-branching, bouton-free segment believed to project to white matter.

TABLE S1. Individual Axon Arbor Results

TABLE S1 (b) BASKET AXON WIRE ECONOMY

axon	cell type	boutons	Original axon arbor total length(mm)	Axon Graph total arbor length(mm) L_t	MST total arbor length (mm) L_{MST}	Wire Economy ratio (axon) $\varepsilon = L_{MST}/L_t$	Excess wire (mm) $(L_t - L_{MST})$	ESMT/MST Steiner Ratio $\rho_3 = L_{ESMT}/L_{MST}$
oi26lbc	basket	5211	44.80	41.36	32.99	0.80	8.37	0.991162
oi38lbc	basket	5020	57.91	50.01	38.10	0.76	11.91	0.990457
oi55rbc2	clutch	3766	35.53	32.36	24.93	0.77	7.43	0.990587
oi28lbc	basket	5676	84.80	72.48	53.45	0.74	19.03	0.988690
oi57lbc1	basket	5394	44.05	42.08	33.28	0.79	8.80	0.994704
oi57lbc2	basket	7070	59.27	56.43	42.78	0.76	13.65	0.991648
oi57lbc3	basket	4894	53.32	51.06	38.06	0.75	13.00	0.990939
oi57lbc4	basket	5624	48.94	48.56	37.41	0.77	11.15	0.992341
oi57rbc1	basket	1409	18.70	18.83	13.79	0.73	5.04	0.988034
mean	-	4919	49.95	43.39	33.06	0.76	10.33	0.99
SD	-	1582	18.66	15.08	11.09	0.02	4.13	

TABLE S1. Individual Axon Arbor Results

TABLE S1 (c) SPINY AXON PATH ECONOMY

axon	*cell type*	*boutons*	*Axon average path length (mm)* P_{axon}	*MST average path length (mm)* P_{MST}	*SPT average path length (mm)* P_{STAR}	*Path Economy ratio (axon)* $\gamma = P_{STAR}/P_{axon}$	*Path Economy ratio (MST)* $\gamma = P_{STAR}/P_{MST}$
oi15rpy4	pyramidal	1879	1.88	2.58	1.33	0.71	0.52
oi24rpy1	pyramidal	1046	0.89	1.37	0.68	0.76	0.49
oi27rpy1	pyramidal	3949	1.09	1.78	0.72	0.66	0.40
oi27rpy2	pyramidal	2183	0.93	1.93	0.67	0.72	0.35
oi28rpy1	pyramidal	6184	1.82	5.52	1.32	0.73	0.24
oi33lpy1	spiny stellate	1348	0.81	1.08	0.48	0.59	0.44
oi53rpy1	pyramidal	1232	1.32	1.51	0.81	0.61	0.54
oi57rpy1	pyramidal	1844	0.77	1.54	0.45	0.59	0.29
oi57rpy2	pyramidal	1295	0.84	1.08	0.55	0.65	0.51
oi57lpy6	pyramidal	1429	0.95	1.99	0.66	0.69	0.33
mean	-	2239	1.13	2.04	0.77	0.67	0.41
SD	-	1618	0.41	1.30	0.31	0.06	0.10

TABLE S1 (d) BASKET AXON PATH ECONOMY

axon	*cell type*	*boutons*	*Axon average path length (mm)* P_{axon}	*MST average path length (mm)* P_{MST}	*SPT average path length (mm)* P_{STAR}	*Path Economy ratio (axon)* $\gamma = P_{STAR}/P_{axon}$	*Path Economy ratio (MST)* $\gamma = P_{STAR}/P_{MST}$
oi26lbc	basket	5211	0.38	0.79	0.25	0.66	0.31
oi38lbc	basket	5020	0.46	0.92	0.29	0.63	0.31
oi55rbc2	clutch	3766	0.48	0.89	0.24	0.50	0.27
oi28lbc	basket	5676	0.85	1.77	0.64	0.75	0.36
oi57lbc1	basket	5394	0.60	1.05	0.42	0.70	0.40
oi57lbc2	basket	7070	0.64	1.31	0.41	0.64	0.31
oi57lbc3	basket	4894	0.77	1.54	0.47	0.61	0.30
oi57lbc4	basket	5624	0.69	1.30	0.50	0.72	0.39
oi57rbc1	basket	1409	0.66	1.12	0.47	0.71	0.42
mean	-	4919	0.61	1.19	0.41	0.66	0.34
SD	-	1582	0.15	0.32	0.13	0.07	0.05
